# The Evolving Security of the Internet of Vehicles: A Survey from Classical Machine Learning to Large Language Models, Adversarial Robustness, and Explainable AI

**DOI:** 10.3390/s26185862

**Published:** 2026-09-16

**Authors:** Meisam Sharifi Sani, Saeid Iranmanesh, Raad Raad

**Affiliations:** Faculty of Engineering and Information Sciences, University of Wollongong, Wollongong, NSW 2522, Australia; mss868@uowmail.edu.au (M.S.S.); raad@uow.edu.au (R.R.)

**Keywords:** Internet of Vehicles, machine learning, intrusion detection, federated learning, blockchain, adversarial robustness, explainable AI, large language models, edge security, CAN bus, vehicular network security

## Abstract

The Internet of Vehicles (IoV) connects vehicles, roadside infrastructure, and cloud or edge platforms to support safer and more efficient transportation. This connectivity also exposes vehicular networks to message spoofing, denial-of-service, and man-in-the-middle attacks, and to threats that target the machine-learning defenses themselves, such as model poisoning, gradient inversion, and adversarial examples. Machine learning and deep learning are now the dominant defensive tools, and the resulting literature is large and fragmented. This survey reviews that literature using a documented database search and stated inclusion criteria. The retained studies are organized into three categories, each defined by the dominant security-design problem it addresses. The categories cover classical detection and prevention, decentralized security based on federated learning and blockchain, and the assurance directions of Large Language Model (LLM)-driven detection, adversarial robustness, and explainable artificial intelligence. Rather than pooling reported scores, the survey records the evaluation setting of each study. This shows that de-duplication, class balancing, and metric aggregation account for much of the apparent variation in reported performance. A study-level assessment finds that no reviewed study demonstrates more than one of the three assurance capabilities, and no detector evaluated on vehicular traffic has been tested against adversarial perturbation. The survey concludes with a cross-category comparison, an EU AI Act alignment assessment, and recommendations for future research.

## 1. Introduction

The rapid advancement of wireless communication, sensing, and computing technologies has transformed modern vehicles from isolated transportation units into connected and intelligent cyber–physical systems [1]. This transformation has given rise to the Internet of Vehicles (IoV), a paradigm in which vehicles, roadside infrastructure, cloud platforms, and other road users exchange information to enhance road safety, traffic efficiency, and driving comfort [2].

Early vehicular communication relied on Vehicular Ad hoc Networks (VANETs), which enabled short-range communication between vehicles and between vehicles and infrastructure [3]. While VANETs established the technological foundation for vehicular networking, their practical deployment was constrained by dynamic topologies, intermittent connectivity, and limited processing capability [4,5,6]. With the emergence of advanced cellular and edge computing technologies, vehicular networking has evolved beyond VANETs to the more comprehensive and data-driven IoV paradigm [7].

**Figure 1:**
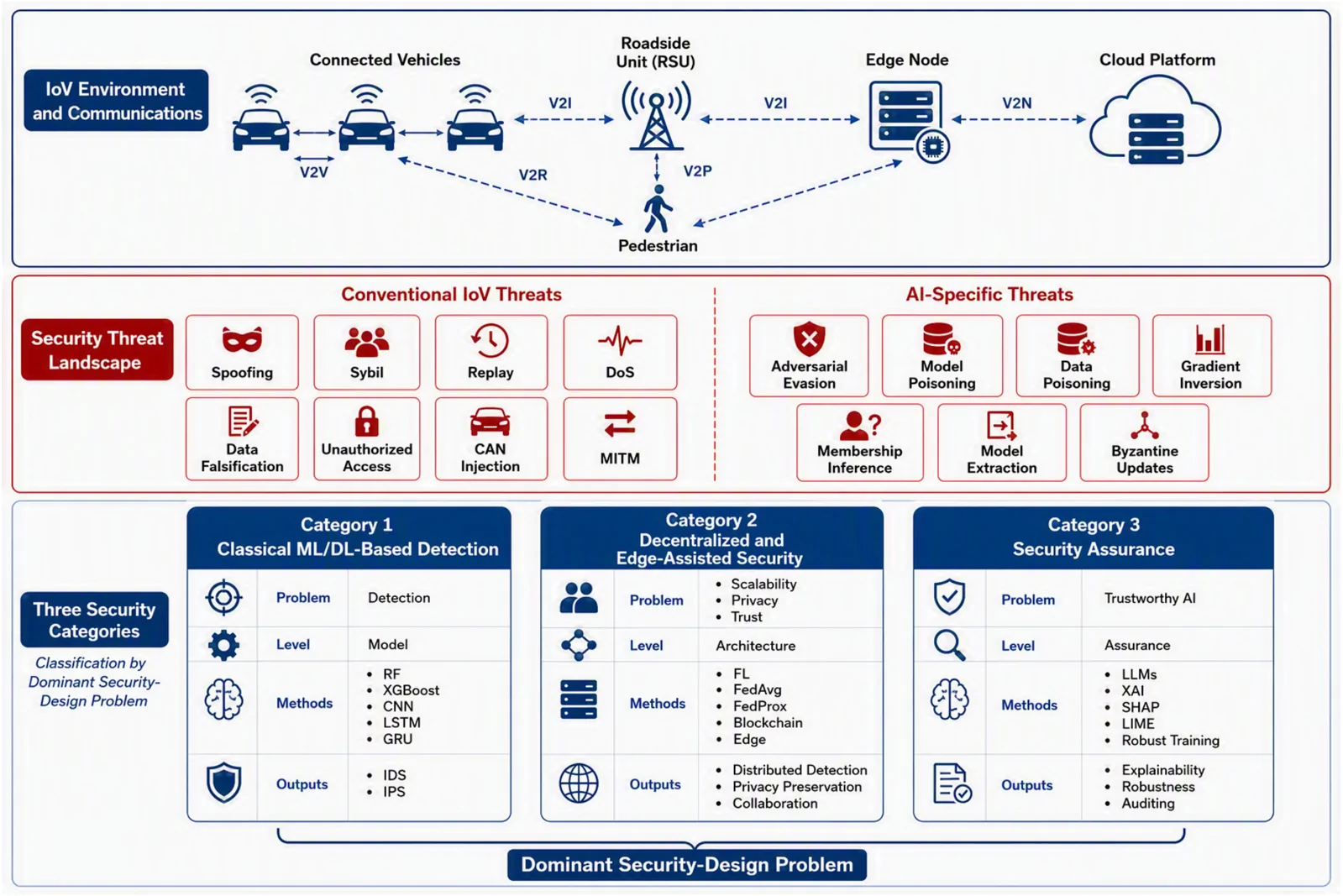
An analytical map of IoV communications highlights key security threats and the three security categories used in this survey.

Unlike VANETs, IoV integrates heterogeneous communication technologies and intelligent computing to support Vehicle-to-Everything (V2X) interactions, as illustrated in Figure 1. These interactions enable advanced applications such as autonomous driving, cooperative traffic management, and intelligent navigation. However, the openness, connectivity, and real-time requirements of IoV substantially expand the attack surface, making security a persistent and evolving challenge.

IoV networks remain highly susceptible to threats such as spoofing, replay, Denial-of-Service (DoS), data falsification, and unauthorized access, which can compromise data integrity, driver privacy, and physical safety [8]. Traditional rule-based and cryptographic defenses are often insufficient because of their limited adaptability and their inability to cope with unknown or evolving attacks under highly dynamic vehicular conditions. A further and increasingly recognized class of threats targets the machine-learning-based defenses themselves: an adversary who understands a detector’s structure can craft inputs specifically designed to evade it [9,10], poison training data or model updates to corrupt a shared federated model [11], or infer sensitive information from gradient updates [12].

Machine learning has therefore emerged as a central enabler for IoV security, capable of learning patterns from large-scale vehicular data and adapting to conditions that static defenses cannot anticipate [13]. The security problems this role has to address are, however, of different kinds. One body of work concentrates on the detection effectiveness of classical machine-learning and deep-learning classifiers; a second addresses decentralized, privacy-preserving deployment at the network edge; and a third is concerned with the assurance of the detector itself, through language-model-driven analysis, adversarial robustness and interpretability [10,14]. These concerns are pursued concurrently rather than in succession. The shifts in the field prompt us to organize the information not as a simple list of techniques, but into three distinct categories. Each category is defined by the main security-design problem it addresses and the primary level at which its technical interventions take place. These three categories are present in the existing literature, and the criteria for assigning a study to one of them are explained in Section 3.4.

### 1.1. Related Surveys

The surveys most relevant to this work identify machine learning as a key enabling technology for the security of the IoV or the Internet of Things (IoT). However, each survey organizes its reviewed literature around different themes, and none adopts the primary security-design problem outlined in this study. Three organize around platform or application layer rather than security concern: ref. [15] surveys AI-based techniques for UAV-assisted IoV, and the authors themselves note that security and data privacy are not considered in that work; ref. [16] surveys edge-intelligence approaches for intelligent transport systems around performance, deployment and systems integration, explicitly distinguishing this scope from prior work that focused on security among other specific technologies; and ref. [17] organizes IoV security around the integration of sensing, communication, computing and intelligence layers rather than around what each reviewed mechanism is designed to assure, and engages minimally with adversarial robustness and not at all with explainability. Three further surveys organize around a single technique family in isolation from the others: ref. [18] reviews deep-reinforcement-learning intrusion detection across general IoT deployments, from household to industrial equipment, without a vehicular focus or engagement with decentralized or explainability-oriented methods; ref. [19] reviews machine-learning intrusion detection covering both intra-vehicle and inter-vehicle communication in connected and autonomous vehicles, without decentralized edge mechanisms, large language models, or explainability; and ref. [8] treats trust management as a dedicated pillar, with a detailed taxonomy of trust properties, metrics and computation, but does not extend that treatment to federated-learning-based or blockchain-coordinated trust mechanisms, which receive only a single passing mention among traditional approaches. Read together, these surveys show that the technical building blocks of IoV security, namely classical detection, decentralized coordination, and language-model-driven assurance, are individually well recognized, but to the best of our search, no existing survey groups them by the security-design problem each is meant to solve.

### 1.2. Comparison of Existing Surveys and This Work

Table 1 compares the platform and technical scope of the surveys discussed above with the proposed work, organized around the specific families of machine-learning techniques each survey addresses. As the last three columns show, large language models, adversarial robustness, and explainable AI are not jointly addressed by any prior survey of IoV security, including the two most recent publications in this space.

Organizing by dominant security-design problem, rather than by the technique family used for comparison above, keeps these three concerns analytically distinct even though they are pursued concurrently and frequently share techniques, as Section 3.4 establishes. A technique-family taxonomy records whether a method family is covered; it cannot show which design problem a given use of that technique is meant to solve, since the same technique can serve more than one problem depending on how it is deployed. Organizing by design problem instead makes it possible to ask, within a single category, whether the specific capabilities that problem requires are jointly demonstrated by any one study, rather than merely present somewhere in the literature.

### 1.3. Contributions

The main contributions of this paper are summarized as follows:

- We introduce a three-category classification of machine-learning-driven security research in the IoV, and state explicitly the single criterion by which each category is defined.
- We consolidate Category 1 research on classical machine learning and deep learning for intrusion detection, recording the evaluation setting of each study so that figures are compared only where the evaluations are comparable.
- We examine Category 2 decentralized mechanisms, covering federated learning with its non-IID data, Byzantine robustness, and differential privacy trade-offs, together with blockchain-assisted trust management.
- We present one of the first integrated treatments of the Category 3 directions for the IoV, covering language-model-driven detection, adversarial robustness with a structured attack taxonomy, and explainable AI.
- We provide a cross-category comparison table and a study-level gap analysis scoring each Category 3 study on contextual analysis, demonstrated robustness, and explainability.
- We document the literature selection methodology, namely the database searched, the query executed, the time window, the selection criteria, the records retained at each stage, and the limitations of the resulting corpus.

### 1.4. Literature Selection Methodology

A structured methodology was employed to compile the literature reviewed in this survey, focusing on machine learning approaches to security within the IoV. The search combined relevant vehicular terms, such as “Internet of Vehicles”, “VANET”, “connected vehicle”, “CAN bus” and “V2X”, with security-related terms, such as “intrusion detection”, “misbehaviour detection” and “trust management”, and with methodological terms, such as “machine learning”, “federated learning”, “blockchain”, “large language models”, “adversarial” and “explainable AI”. A supplementary search paired vehicular terms with specific emerging-method keywords, including large language models, adversarial examples, certified robustness, and explainability methods. This additional query was necessary because studies in these emerging areas are often indexed by the method itself rather than by broader security terms such as intrusion detection or trust management. The database used for this research was Scopus, which indexes key venues such as IEEE, ACM, Elsevier, Springer, and MDPI where vehicular security research is published. The publishing date range selected was from 2018 to 2026, chosen because vehicular security research applying machine learning begins to appear in volume from 2018 onward. This range governs the studies retrieved by the query rather than the whole reference list: the foundational methods on which Category 3 work builds were published between 2015 and 2020, and they were reached through citation tracking from the retained studies rather than through the query itself. The executed query and the number of records remaining after each successive filter are provided in Table 2.

The selection criteria were established to ensure that the review concentrated on assessed security mechanisms. A study was included if it proposed or evaluated a security mechanism for a vehicular system, provided an evaluation instead of a simple architectural description, and addressed a security design problem that falls within one of the three categories outlined in Section 3.4. Studies focused on vehicular networking that did not have a security objective were excluded, along with those that duplicated earlier reports by the same authors or did not provide any evaluation. The studies retained were thoroughly reviewed in full, rather than relying on abstracts. Every value reported in the summary tables was transcribed from the primary source and independently checked by a second author. Additionally, backward and forward citation tracking from the retained studies provided the foundational methods described in Section 6, as well as the datasets, standards, and regulations referenced throughout the work.

Screening was conducted in two stages. Records were prioritized based on their relevance as determined by the database, and their titles and abstracts were evaluated against the inclusion criteria, resulting in 170 candidate records. The screening process was purposive rather than exhaustive, and backward and forward citation tracking from the retained studies was employed to minimize the risk of missing relevant work. The full text of 60 of these records was obtained and assessed for eligibility, while the others were excluded after a closer examination of their abstracts. The 34 studies constitute the primary evidence corpus used for the study-level analysis and comparison in Section 4, Section 5 and Section 6: nine in Category 1 (Table 6), twelve in Category 2 (Table 8), and thirteen in Category 3 (Table 11 and Section 6.3). The remaining 56 of the 90 references in the bibliography serve as foundational, methodological, dataset, standard, regulatory, or contextual sources and are not treated as primary evidence in the study-level synthesis.

### 1.5. Paper Organization

**Figure 2:**
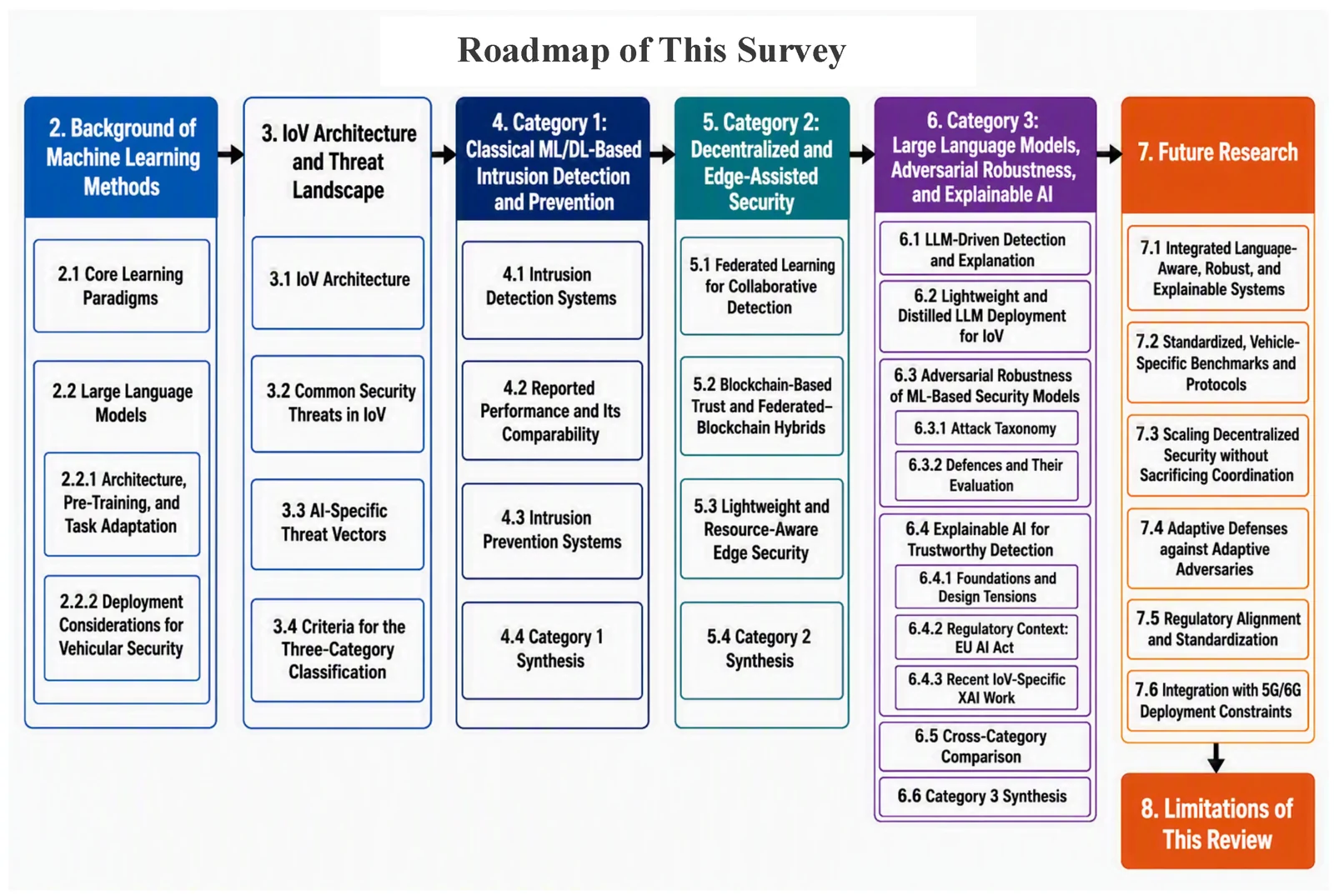
Hierarchical roadmap of the survey.

The remainder of this paper is organized as follows. Figure 2 shows how this survey is organized. Section 2 introduces background machine-learning concepts. Section 3 describes the IoV architecture and threat landscape, including intra-vehicle networks and AI-specific threats. Section 4 reviews the Category 1 classical ML/DL-based intrusion detection and prevention. Section 5 discusses the Category 2 decentralized and edge-assisted security. Section 6 presents the Category 3 security directions and a cross-category comparison. Section 7 outlines open challenges and future research directions, and Section 9 concludes the paper.

## 2. Background of Machine Learning Methods

Machine learning underlies most of the techniques discussed later in this paper, so this section briefly introduces the main learning paradigms and their positioning for vehicular network security. The exception is Section 2.2, which treats large language models in greater depth than the other paradigms, because this class of model is central to the Category 3 security directions and introduces considerations of transformer architecture, hallucination risk, and inference cost that the classical paradigms do not raise.

### 2.1. Core Learning Paradigms

Supervised learning trains a model on labeled data so that it can predict outcomes for new, unseen inputs. It is commonly divided into classification, which predicts discrete categories, and regression, which predicts continuous values. In vehicular security, it is widely used to distinguish normal traffic from known attack patterns and to identify specific types of misbehavior [20].

Unsupervised learning works with unlabeled data to uncover hidden structure, typically by grouping similar observations into clusters. Because labeled attack data is often scarce in real vehicular deployments, unsupervised methods are particularly useful for surfacing previously unseen misbehavior that supervised models would miss [20].

Reinforcement learning trains an agent to make sequential decisions by interacting with an environment and receiving scalar rewards. This learning style suits vehicular settings, where conditions change continuously and a fixed pre-trained model may quickly become outdated [18].

Deep learning uses multi-layer neural networks to learn hierarchical representations directly from raw data, reducing the need for manual feature engineering. Its capacity to model complex, non-linear relationships has made it a common choice for intrusion detection, where traffic volume and complexity continue to grow [21].

FL enables multiple vehicles or edge nodes to collaboratively train a shared model while keeping raw data local, exchanging only model updates with a coordinating server. The canonical protocol is FedAvg [22], which averages client gradients weighted by local dataset size. This design reduces communication overhead and preserves the privacy of vehicle and driver data [23].

Transfer learning reuses knowledge gained from a previously learned task to enhance learning on a new, related task. In vehicular security, this is valuable when labeled attack data for a specific network or vehicle type is limited, allowing detection models trained elsewhere to be quickly adapted [24].

### 2.2. Large Language Models

#### 2.2.1. Architecture, Pre-Training, and Task Adaptation

Large Language Models (LLMs) are built on the transformer architecture introduced by Vaswani et al. [25], whose core component is multi-head self-attention. Unlike recurrent networks, the attention mechanism computes relationships between all positions in a sequence simultaneously, giving transformers superior capacity to capture long-range dependencies without the vanishing-gradient problem that limits recurrent architectures. Each attention head independently learns a different relational pattern, and the outputs are concatenated and projected, allowing the model to attend to different aspects of the input sequence in parallel. This property is directly relevant to vehicular network security: a sequence of CAN-bus frames or V2X messages contains temporal dependencies that span many time steps, and single-snapshot classifiers miss precisely those dependencies that attention can capture.

Modern LLMs are pretrained on very large corpora using one of three principal objectives: masked language modeling (BERT-style, where randomly masked tokens are predicted from context [26]), causal language modeling (GPT-style, where each token is predicted from preceding tokens [27]), and sequence-to-sequence objectives that train an encoder–decoder architecture. Pretraining on extensive text corpora provides LLMs with a valuable prior on sequential structure that can be adapted to other sequential domains such as network logs and protocol message sequences through fine-tuning on a smaller, in-domain dataset.

After pretraining, an LLM is adapted to a specific task either by fine-tuning (updating all or a subset of parameters on labeled in-domain data) or by prompt engineering (framing the task as a natural-language query that the frozen pre-trained model can answer without parameter updates). Both approaches are being explored for the detection of network intrusion. Fine-tuning requires labeled attack examples but produces a model specialized to the target domain; prompt engineering is more flexible but may produce less precise classifications when the task is highly specialized.

#### 2.2.2. Deployment Considerations for Vehicular Security

Three practical factors govern whether an LLM-based detector can be deployed on vehicular hardware: its tendency to hallucinate, the way network traffic must be represented as tokens, and the computational cost of inference.

Language models often generate fluent output that may not accurately reflect the input or be factually supported. This issue has been examined for natural-language generation by Ji et al. [28] and specifically for large language models by Huang et al. [29]. It is measurable rather than anecdotal: on the TruthfulQA benchmark, the best model evaluated answered truthfully on 58% of the questions, compared to 94% for human respondents [30]. The behavior persists in safety-critical perception, where vision-language models have been observed to answer from general knowledge or textual cues rather than from visual grounding, particularly when the visual input is degraded or missing [31]. The issues are evident in the vehicular literature reviewed here. For example, the natural-language rationale generated by the CAN-bus detector developed by Quintano et al. [32] incorrectly identifies the length code as part of the payload. Additionally, the authors note inconsistencies in the use of field names within the generated explanations. In general text applications, errors of this nature are primarily concerns related to accuracy. However, in a security function where the output can trigger an automated response, these errors raise safety concerns. Therefore, establishing boundaries and auditing the confidence of model outputs should be part of the design process for an IoV detector, rather than being addressed only during its evaluation.

Applying a language model to network data requires converting binary and numerical fields into a token sequence, and the studies reviewed here use four distinct schemes. A record can be transformed into a simple string composed of column names and their corresponding values. This string can then be tokenized using the model’s own vocabulary and padded to a fixed length [33]. Alternatively, field names may be omitted, retaining only the ordered values, which results in a more compact sequence, such as tcp - 0.398803 0 0 REJ T T 0 Sr 1 60 1 40 [34]. Another approach involves concatenating each field with its column name and then hashing the result before tokenization. This method allows the model to learn from fixed-length hash strings while ensuring that the underlying values are not exposed during the training process [35]. Lastly, the fields of a single frame can be mapped to a short, fixed token sequence and incorporated into a natural-language prompt [32]. The choice between different encoding methods is significant: re-encoding the same data flows as key-value pairs instead of a simple sequence of values can lead to a decrease of approximately one percentage point in precision, recall, and F1 score, as noted in [34]. This choice also affects the sequence length, and consequently, the inference cost. For instance, a serialized flow record is padded to 128 tokens in [33], while a single CAN frame is represented by just eleven tokens in [32].

The cost of a transformer forward pass has two parts: a term proportional to the non-embedding parameter count and linear in sequence length, and an attention term quadratic in sequence length [36]. At the short sequences typical of tokenized network traffic, the first term dominates. For BERT-base, with 110 M parameters [26], a forward pass over 128 tokens at batch size one costs approximately 22.5 GFLOPs when a multiply-accumulate is counted as two operations [37], and models at GPT-3 scale require orders of magnitude more. Measured on mobile-class hardware, a single such forward pass takes 342 ms on a four-thread Pixel 4 [38] and 1.69 s on a Pixel 3 [37]. These smartphone figures are used here only as an approximation of the computational burden of an uncompressed, BERT-scale language model; they are not a benchmark of automotive-grade compute platforms. Actual in-vehicle latency depends on the deployed automotive System-on-Chip (SoC), accelerator, inference runtime, and quantization, and should accordingly be read as an order-of-magnitude feasibility indication rather than a direct automotive-hardware benchmark. The timing budget available in the vehicle is far tighter: a standard-format CAN frame carrying eight data bytes occupies 135 bit times in the worst case [39], so a 500 kbit/s bus admits on the order of $3.7\times 10^{3}$ frames per second, leaving well under a millisecond per frame. This comparison is therefore best interpreted as an indicative upper-bound proxy for the computational burden of processing serialized network-traffic windows, rather than as a per-frame latency estimate. Together with the 7–60 W power envelopes of embedded inference modules realistically available for in-vehicle and roadside deployment [40], these results indicate that direct deployment of an uncompressed BERT-scale model remains challenging under tight per-message latency and energy budgets. Knowledge distillation [41], which transfers learned representations from a large teacher model to a compact student model, is the primary pathway for making language-model-style detection compatible with this hardware, and forms the subject of a dedicated subsection in Section 6.

## 3. IoV Architecture and Threat Landscape

### 3.1. IoV Architecture

**Figure 3:**
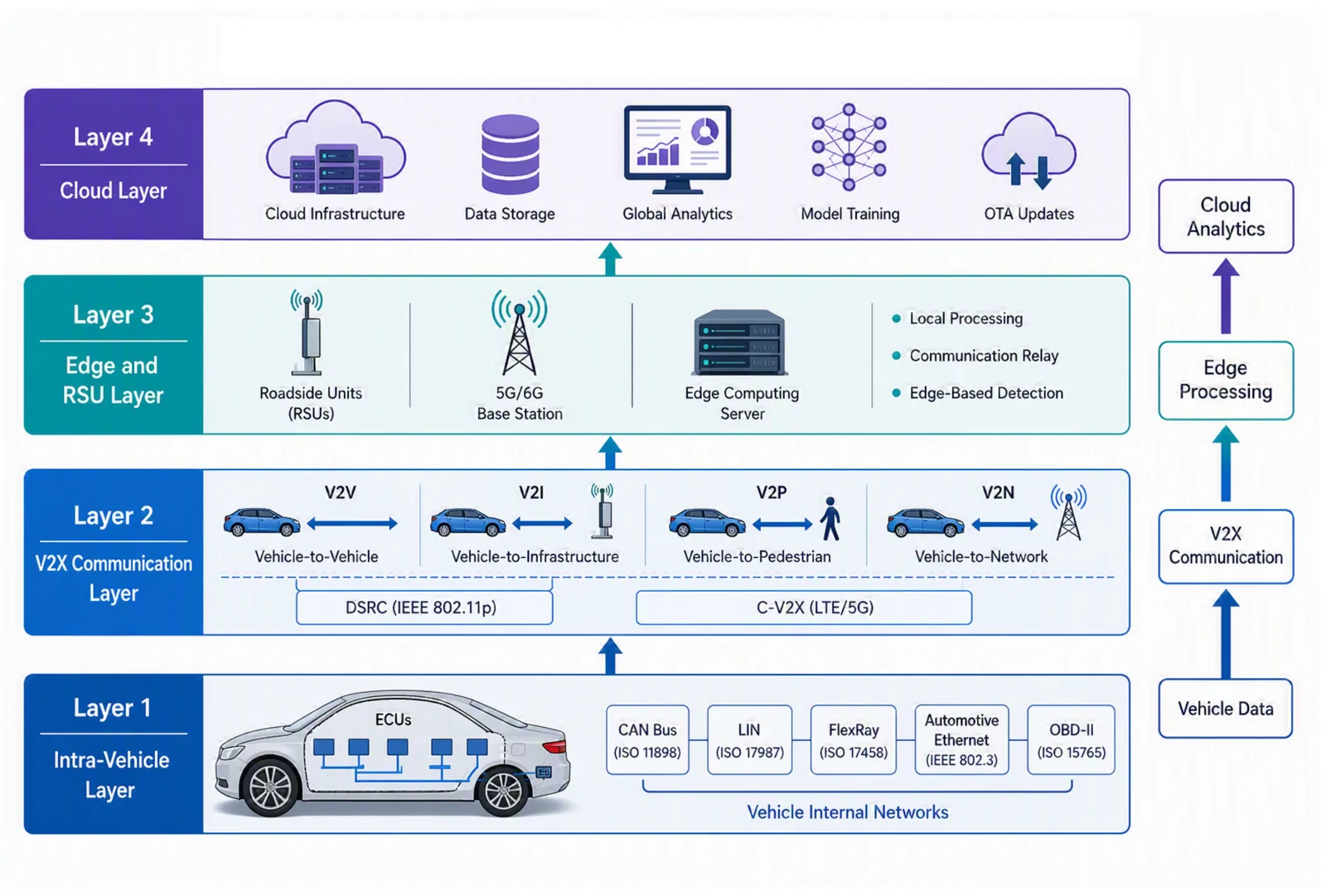
Layered architecture of the IoV, from in-vehicle networks through V2X communication and edge processing to the cloud.

The IoV connects vehicles, road infrastructure, and cloud platforms into a layered network [42], as illustrated in Figure 3. Four principal layers are relevant to this survey’s security framework.

Modern vehicles contain dozens of Electronic Control Units (ECUs) connected by several internal network protocols. The Controller Area Network (CAN bus, ISO 11898) is the most widely deployed, but vehicles increasingly use the Local Interconnect Network (LIN, ISO 17987) for low-speed peripherals, FlexRay (ISO 17458) for safety-critical chassis control, and Automotive Ethernet (IEEE 802.3) for high-bandwidth applications. OBD-II (ISO 15765) provides a standardized diagnostic port that exposes the internal bus to external access. The CAN bus in particular lacks native authentication or encryption, making it the most studied attack surface in the Category 1 literature. Table 3 summarizes the key characteristics of each intra-vehicle protocol.

Vehicles communicate with other Vehicles (V2V), Vehicles-to-Infrastructure (V2I), Vehicles-to-Pedestrians (V2P), and the Vehicles-to-Network (V2N) using either Dedicated Short Range Communication (DSRC, IEEE 802.11p) or Cellular V2X (C-V2X, LTE or 5G NR). Both are broadcast protocols with limited built-in authentication, exposing them to identity and content manipulation.

RSUs and multi-access Edge Computing (MEC) nodes provide local processing, caching, and communication relay. They are responsible for distributing safety messages and increasingly for running edge-based intrusion detection. Their resource constraints are tighter than those of cloud servers, but less extreme than in-vehicle ECUs.

Cloud infrastructure supports long-term data storage, global model training for federated systems, and Over-The-Air (OTA) software updates. While powerful, the cloud introduces latency unsuitable for real-time safety decisions and is a high-value target for large-scale attacks.

### 3.2. Common Security Threats in IoV

The open and real-time nature of vehicular communication subjects the IoV to various network-layer and application-layer threats, as summarized in Table 4 [43]. Spoofing and Sybil attacks manipulate identity or position. DoS attacks exhaust communication or computational resources. Eavesdropping and Man-in-the-Middle (MITM) attacks target data confidentiality. Routing attacks (black-hole, gray-hole, wormhole) misdirect or drop forwarded traffic. Data falsification injects misleading sensor or application data [44].

### 3.3. AI-Specific Threat Vectors

As machine learning becomes central to IoV security, a distinct and increasingly important class of threats targets the ML components themselves. These AI-specific threats are not addressed by network-layer defenses alone and require dedicated treatment.

An adversary with knowledge of a trained detector’s parameters (white-box setting) or its input–output behavior (black-box setting) can craft inputs that are slightly perturbed from normal traffic but are classified as benign by the detector. This class of attack is discussed in detail in Section 6.3, where specific attack methods (FGSM, PGD, C&W) are described.

In federated learning, malicious participants can submit manipulated model updates designed to bias the global model toward producing incorrect outputs for specific inputs. Unlike data poisoning (manipulating training data), model poisoning acts directly on gradient updates, making it harder to detect. A Byzantine adversary [11] controls a subset of federated participants and acts arbitrarily to maximize damage to the shared model while avoiding detection by aggregation protocols.

Recent work has shown that an honest-but-curious federated server can reconstruct training samples from gradient updates with high fidelity [12]. In IoV, this means that exchanging gradients instead of raw data does not necessarily protect vehicle trajectory, speed, or identity information from a server that analyzes updates.

Given access to a trained model, an adversary can determine with above-chance accuracy whether a specific data record was included in the training set. In vehicular deployments, this can reveal whether a particular vehicle’s behavior pattern was used to train a shared intrusion detection model.

By querying a deployed model with carefully chosen inputs and observing outputs, an adversary can build a functionally equivalent surrogate model. This surrogate can then be used to craft adversarial evasion inputs more efficiently, bypassing the black-box constraint.

### 3.4. Criteria for the Three-Category Classification

A work is assigned to a category according to its *dominant security-design problem*: the primary unresolved security requirement that motivates the study and determines its principal technical intervention. The model, system architecture, or assurance level is therefore used as an operational indicator of this problem, rather than as an independent classification axis. Table 5 summarizes the resulting criterion.

Algorithmic families may occur in more than one category and are therefore not sufficient for classification. A CNN or recurrent model used as a centralized CAN-bus classifier is classified as Category 1 because the contribution is primarily model-level detection; the same architecture trained through federated learning across vehicles is classified as Category 2 because the contribution is primarily architectural; and a detector whose principal contribution is adversarial robustness or explainability is classified as Category 3 because the contribution concerns security assurance. When a study addresses multiple concerns, it is assigned according to the problem that the study explicitly foregrounds, with cross-category overlap noted where necessary.

The ordering reflects conceptual dependency rather than publication chronology. Category 1 focuses primarily on improving detection effectiveness through learned classifiers, while leaving centralization, scalability, and data privacy largely unresolved. Category 2 addresses these deployment and coordination constraints through distributed learning, edge-assisted processing, privacy mechanisms, and decentralized trust, while introducing additional challenges concerning coordination, transparency, and assurance. Category 3 addresses assurance requirements associated with adversarial robustness, interpretability, and language-based contextual analysis. This ordering therefore describes how different security-design problems relate to one another; it does not imply that one category replaces another.

The publication years of the reviewed studies overlap almost completely in the three categories. The Category 1 studies in Table 6 were published between 2023 and 2026, the Category 2 studies in Table 8 between 2023 and 2026, and the vehicular Category 3 studies in Table 10 between 2024 and 2026, with a median of 2025 in each case. The three categories therefore coexist in the recent literature, while the eight foundational methods on which Category 3 draws were published between 2015 and 2020, several years before their vehicular application. This delay separates the invention of a method from its adoption in the field and is not evidence of the succession of one category over another.

## 4. Category 1: Classical ML/DL-Based Intrusion Detection and Prevention

Part of the Category 1 literature is evaluated wholly or partly on generic network-intrusion datasets rather than on vehicular traffic. Such studies are retained because they examine detection architectures that are discussed in the IoV security literature or are explicitly motivated by external-network security in connected-vehicle systems; however, they are identified as non-vehicular wherever they appear and are not treated as direct evidence of IoV-specific detection performance.

### 4.1. Intrusion Detection Systems

A first strand of recent work asks how much detection accuracy can be obtained from lightweight, classical models before deep architectures are needed. Tree-based ensembles built for multi-class attack recognition in IoV show that carefully constructed random forests and gradient-boosted trees can separate several attack categories while remaining computationally cheap and comparatively interpretable: on the Car-Hacking dataset the stacked ensemble of [45] reports 99.99% accuracy and an F1 of 0.9999 across five classes. A related line of work confronts class imbalance, the reality that genuine attack traffic is a small fraction of everyday vehicular communication, and shows that ensembles tuned with oversampling strategies such as the Synthetic Minority Over-sampling Technique (SMOTE) can reach perfect macro-averaged scores on CICIoV2024 [46], a result examined further in Section 4.2. Both results argue that classical ensembles remain a serious option rather than a baseline to be dismissed, though they depend on hand-selected features and are, by design, better at recognizing patterns similar to their training data than at generalizing to novel behavior.

A second and more cautionary strand interrogates how results are obtained. An empirical study of CAN-bus intrusion detection shows that once heavily redundant records are removed from a widely used vehicular attack dataset, both lightweight and deep models perform substantially worse than earlier reports suggested: removing duplicate records from CICIoV2024 drives the deep models below a macro-averaged F1 of 0.55 [47], a change quantified in Section 4.2. Some previously celebrated results may therefore reflect a model memorizing repeated records rather than learning a generalizable notion of an attack.

A third strand turns to recurrent and convolutional architectures that model the CAN bus as a stream of related messages rather than a set of independent records. Deep models designed for CAN-bus intrusion detection, among them Long Short-Term Memory networks (LSTMs), Gated Recurrent Units (GRUs) and convolutional variants, can pick up sequence-dependent irregularities that a snapshot-based classifier misses [48]; on a merged corpus of the Car-Hacking, OTIDS and Survival Analysis traces the LSTM reaches 99.89% accuracy on the binary task, while the multi-class results are reported one class at a time rather than as an aggregate. The trade-off is familiar: these architectures require more computation and are harder to interpret than tree-based ensembles, so their advantage is most justified when attacks genuinely depend on message ordering or timing rather than on the content of any single message.

A fourth strand builds detection as a pipeline rather than a single model. One recent framework combines feature selection with an ensemble of classifiers to secure cloud-assisted VANETs, aiming to keep detection reliable as the network scales [49]; its evaluation, however, is performed on CICIDS2017, whose underlying traffic is generated in an enterprise-network testbed rather than in a vehicular environment. A stacked design for connected vehicles addresses both internal and external networks [50]. The internal component, tested with vehicle CAN-bus data in a CANoe environment, achieves an accuracy of 99.99% and an F1 score of 0.999 across nine classes. The external component, evaluated on the CICIDS2017 dataset, reports an F1 score of 0.9977; however, this score drops to 0.9467 when deployed on a Raspberry Pi. This indicates that the impressive accuracy observed in testing may not be fully replicated when implemented on in-vehicle hardware. For these studies, the evaluation settings and datasets are summarized in Table 6 before their reported results are compared.

Similarly, the tree-based ensemble study discussed above [45] evaluates both an external network-intrusion setting using CICIDS2017 and an in-vehicle setting using the Car-Hacking dataset; these two evaluation domains are therefore treated separately in Table 7. Extending coverage across attack surfaces comes at the cost of pipeline complexity: more components that must be individually maintained, retrained, and kept mutually consistent.

A fifth strand shows that detection is increasingly paired with cryptographic authentication rather than deployed as a standalone layer. A framework for V2X communication pairs Elliptic Curve Digital Signature Algorithm (ECDSA)-based authentication with ML-based anomaly detection, on the grounds that cryptography and behavioral detection address complementary failure modes: tampering and impersonation on the one hand, subtle misbehavior on the other [51]. That study reports 98.2% for privacy preservation and 95.7% for processing efficiency but no classification accuracy, precision, recall or F1, so it contributes a design pattern rather than a detection result. This hybrid framing bridges naturally into prevention, since detection and response are treated as two parts of the same design problem.

It is worth closing this subsection with a limitation that cuts across every method discussed above: none of them is designed with an adversary who deliberately manipulates the model’s input in mind. Recent work shows that detectors which otherwise perform well can be misled by inputs specifically crafted to evade them [10]. Strong reported detection accuracy does not, by itself, guarantee resilience against a motivated attacker. This concern is revisited directly in Section 6.3.

### 4.2. Reported Performance and Its Comparability

Table 6 records the evaluation setting that each primary source states before any figure is quoted. Of the seven, two report no F1, precision or recall at all; five report an F1 value, but only two state how the headline figure is aggregated; one applies de-duplication; and two apply resampling before partitioning, so that their evaluation sets are not the distributions on which the remaining studies test. Table 7 then presents the two benchmarks on which more than one study reports a result. The two studies on CICIoV2024 evaluate comparable versions of the same benchmark and both report macro-averaged metrics, making them substantially more comparable than the studies on CICIDS2017. The three studies on CICIDS2017 do not, and the columns of that table record what differs between them. The four remaining results, each the only one reported on its dataset, are given with their figures in Section 4.1 rather than tabulated, since no cross-study comparison is available for them.

The two studies on CICIoV2024 report macro-averaged F1 of 1.00 and 0.6415 on the same benchmark. This discrepancy is not due to the data itself. The study by [46] does not remove duplicate records and evaluates all 1,408,219 traces using a stratified 80/20 split with 10-fold cross-validation. The study by [47] removes duplicate CAN frames, which reduces the dataset by 99.75% to 3568 unique signatures, and evaluates those using stratified *k*-fold at k=2; its authors attribute the near-perfect figures reported elsewhere on this benchmark to the leakage that such redundancy produces. De-duplication also eliminates one attack class outright, since the STEERING spoofing frames are bit-identical to benign traffic and leave no unique signature, so the two studies classify into six and five classes, respectively. Both, nevertheless, report accuracy above 99%, because benign traffic dominates the evaluation partition in each. On this benchmark, one preprocessing decision therefore moves the macro F1 by 0.36 while moving the accuracy by 0.2 percentage points, which is the clearest available demonstration that accuracy is the weaker of the two measures here.

The three studies on CICIDS2017 cannot be read in the same way, because they do not report a common quantity. They classify into six classes, seven classes, and a number the third does not state; they partition the data 60/40, 70/30, and by a stratified *k*-fold whose *k* is not given; and they differ in whether resampling reaches the evaluation partition, since [45] balances the training set alone while [50] expands the Infiltration class from 36 records to 10,000 before partitioning. Neither of the two that report an F1 value states how it is aggregated, and the third reports no F1, precision or recall at all. The spread of reported accuracy across these three, from 96.2% to 99.90%, therefore measures the difference between three evaluation designs rather than between three detectors. Table 6 shows that this pattern is general rather than isolated: across the seven studies, only one removes duplicate records, two describe a validation protocol without stating its parameters, and only two state how their reported F1 is aggregated.

The CICIDS2017 results also sit outside the vehicular evidence base of this survey, although the studies that report them are within its scope. The dataset was generated on an office network of workstations, web servers, and domain controllers, and its benign class was synthesized from the profiled HTTP, HTTPS, FTP, SSH, and email behavior of twenty-five users. That benign class, rather than the attack classes, is the more consequential mismatch: a detector’s ability to separate office user traffic from an intrusion carries no information about its ability to separate benign CAN frames from injected ones. The attack ontology differs as well. Of the classes the dataset labels, denial-of-service and its distributed variant map onto a threat in Table 4. Brute force against FTP and SSH, web attack, infiltration, botnet, Heartbleed, and port scanning target application-layer services that are not carried on the in-vehicle buses or the V2X links the detectors in this section monitor. These results are therefore evidence about the transferability of the methods rather than about detection in the IoV, and they are excluded from any claim this survey makes about the vehicular evidence base.

### 4.3. Intrusion Prevention Systems (IPSs)

Where the methods above stop at flagging suspicious activity, a growing body of work asks what should happen immediately afterward, increasingly by folding the response into a learned model rather than leaving it to a human operator. One approach embeds a deep learning detector directly into a real-time mitigation loop for the automotive CAN bus, so that a detected anomaly can trigger an automated defensive action within the same operational cycle [52]. The benefit is a much shorter detection-to-response gap, which matters when a delayed response can be as damaging as no response at all; the corresponding risk is that a false detection now has the power to trigger an unwanted action rather than simply an alert.

A more deliberate approach frames the choice of response itself as a decision problem to be learned. A recent intrusion response system uses reinforcement learning together with an explicit uncertainty model to select among candidate defensive actions based on the type of intrusion detected, rather than applying the same countermeasure regardless [53]. This allows the response to be matched more closely to the situation, but the quality of the eventual response still depends entirely on the timeliness and reliability of the feeding detector: prevention amplifies good detection rather than substituting for it.

### 4.4. Category 1 Synthesis

Four points follow from the two subsections above. First, the choice between lightweight classical models and deeper sequence-aware models depends on whether an attack is visible in a single message or only across a sequence, so neither family dominates the other. Second, how a method is evaluated matters as much as how it is designed. On the one benchmark where two studies report the same quantity, a single preprocessing decision moves the macro F1 by more than a third while leaving accuracy almost unchanged. Third, prevention is increasingly designed jointly with detection rather than added after it, which raises the cost of every false positive. Finally, none of the studies whose evaluation settings are recorded in Table 6 reports an evaluation against deliberately crafted inputs. This omission matters for the remainder of the paper, since such inputs directly undermine the premise that a well-performing detector is a trustworthy one, and it motivates Section 6.3.

## 5. Category 2: Decentralized and Edge-Assisted Security

The methods reviewed in the previous section mostly assume a detector has direct access to the data it needs and answers to a single point of authority. That assumption becomes harder to sustain as the number of connected vehicles grows: sending every observation to a distant server introduces latency that safety-relevant decisions cannot tolerate, and pooling raw data from many vehicles raises privacy and trust concerns. The second category of IoV security research responds by pushing computation toward the network edge and by rethinking how distributed nodes can learn and cooperate without fully trusting a central authority.

The most direct response to the latency problem is to move decision-making physically closer to the vehicle. A recent framework for vehicular edge-cloud networks uses deep reinforcement learning to decide in real time how computation should be offloaded between vehicle, edge, and cloud, treating energy efficiency and security jointly [54]. A complementary design combines a deep learning detector with federated coordination directly at the edge of a 5G-capable network, so that detection adapts to local conditions instead of relying on a single globally trained model [55]. The insight connecting both efforts is that edge placement is not simply a matter of running the same model on smaller hardware: decisions about what to compute locally, what to send onward, and how to keep detection current become part of the security design itself. The corresponding limitation is that edge nodes are more resource-constrained and numerous than a central server, so any gain in responsiveness must be weighed against the complexity of keeping many distributed decision points current and consistent.

### 5.1. Federated Learning for Collaborative Detection

Moving detection to the edge raises an immediate follow-up question: how should many edge nodes and vehicles, each seeing only a partial view of the network, learn a shared notion of what an attack looks like without pooling raw data? FL has become the default answer, and a recent taxonomy of federated intrusion detection for the IoV lays out just how varied the resulting designs already are [56].

A critical and frequently under-emphasized challenge in vehicular FL is that vehicles do not see identically distributed traffic: an ambulance, a freight truck, and a commuter car generate fundamentally different data profiles. This non-independent and identically distributed (non-IID) condition causes local models to drift away from one another during training, so a naively averaged global model (as in FedAvg [22]) tends to underperform for any individual vehicle. One line of work responds by personalizing the shared model to each vehicle’s own traffic patterns using a lightweight architecture, producing per-vehicle models that outperform a single global model on heterogeneous fleets [57]. FedProx [58] addresses the same problem by adding a proximal regularization term to local objectives, limiting how far local updates can drift from the global model, and has been adopted in several vehicular FL designs.

FL faces vulnerabilities from Byzantine adversaries, which are malicious clients that submit altered gradient updates intended to poison the global model [11]. The canonical defense is Krum, a robust aggregation rule that scores each client update by its summed distance to its n−f−2 closest neighbors (where *f* is the assumed number of Byzantine clients) and selects the update with the lowest score, rather than averaging all updates. Trimmed Mean [59] provides a complementary approach by discarding the highest and lowest gradient values coordinate-wise before averaging. Neither approach has yet been systematically benchmarked in vehicular FL deployments, representing a gap in the current literature.

Differential privacy (DP) provides a formal guarantee that individual vehicle data cannot be inferred from shared gradient updates by adding calibrated Gaussian or Laplacian noise to each update before transmission. The privacy guarantee comes at a cost: the noise degrades model accuracy, and the trade-off between privacy budget ε and detection utility has been studied in general FL settings but remains insufficiently characterized in the vehicular security domain, where even small accuracy degradations can have safety consequences.

In horizontal FL (the default in most vehicular designs), all participants share the same feature space but different data samples, for example, vehicles of the same type from different cities. In vertical FL, participants share overlapping data samples but have different feature spaces. For example, a vehicle’s onboard sensors and a roadside camera jointly observe the same junction. Vertical FL is relevant in IoV settings where infrastructure and vehicles each hold complementary sensor data about the same events, but its security properties and practical deployment constraints have received little attention in this domain.

As the number of federated participants grows, communication between clients and the coordinating server becomes a bottleneck. Gradient compression (sparsification and quantization), asynchronous aggregation, and local pre-aggregation at edge clusters are active research directions in general FL, but systematic evaluation of these techniques for vehicular intrusion detection, where timely model updates affect safety, has not yet been reported.

A related design integrates federated coordination with a sequence-aware network tailored to in-vehicle traffic, capturing temporal patterns while keeping training local to each vehicle [60]. This study uses an extended version of the publicly available VeReMi dataset to train and evaluate various neural network architectures, including Multilayer Perceptrons (MLPs), GRUs, and LSTM networks.

Explicitly stated, the input, output, and deployment point vary across these designs rather than following a single template. In the LSTM-based design from [60], each vehicle’s local model inputs its position, speed, and one to five preceding messages for temporal context, omitting acceleration and heading due to dataset constraints. The model outputs a probability distribution over eight attack types and a normal class. Each vehicle trains its local model and sends only the model weights to a central server for aggregation. The personalized design described above [57] transforms nine tabular traffic features collected over a sample window into a fixed-size image. It then applies a convolutional bottleneck, which reduces the output to a single sigmoid score that distinguishes between attack traffic and normal traffic. Training occurs at each vehicle, and only encrypted model parameters are exchanged with a cloud server. This server aggregates the data to return a global model, ensuring that there is no edge tier between the vehicle and the server in this design. The taxonomy of federated IDS designs for the IoV categorizes this variability across eight dimensions. These dimensions encompass how data is partitioned, how models are aggregated, and how privacy is enforced. The taxonomy emphasizes that low latency is indispensable for enabling timely reactions in dynamic driving scenarios, where milliseconds can mean the difference between safety and danger [56]. However, in line with the individual designs reviewed earlier, it does not provide a quantitative latency figure for any specific architecture. Read together with the edge-cloud offloading designs discussed earlier in this section [54,55], these studies show that a federated design can aggregate directly at a cloud server with no edge tier, place an edge layer between vehicle and server, or, as with FedAvg [22] and its Byzantine-robust variants [11,58,59] in the abstract, leave deployment location unspecified at the algorithm level; stating this explicitly for each study is necessary because the aggregation algorithm alone does not fix it.

### 5.2. Blockchain-Based Trust and Federated–Blockchain Hybrids

FL solves the data-sharing problem but opens a different one: once many vehicles or edge nodes each contribute model updates, something has to establish that those contributions, and the nodes making them, can be trusted. Blockchain-based designs are the most common answer. A double-layer blockchain architecture separates trust evaluation from data exchange into two coordinated layers, keeping trust computation efficient as more vehicles join [61]. A related design uses an adaptively adjusted trust model built on a permissioned blockchain, so that a vehicle’s standing rises or falls gradually as its behavior is observed over time [62]. The insight shared by both designs is that trust in a decentralized vehicular network cannot be a one-time judgment; it must be continuously computed and recorded in a way that other nodes can verify without relying on a central authority. The limitation is equally consistent: maintaining a shared, tamper-resistant ledger across many fast-moving vehicles adds communication and computational overhead that a fully centralized trust authority would not incur.

FL and blockchain address two complementary weaknesses of decentralized security, namely safe data sharing and trust establishment, so combining them is a natural next step. A recent framework pairs blockchain with FL specifically to secure collaborative intrusion detection, using the ledger to record and validate model updates so that a compromised participant cannot silently corrupt the shared model [23]. A related approach combines blockchain with federated Q-learning, so that decentralized decision-making is coordinated through the same tamper-resistant ledger [63]. A further extension brings a digital twin into this picture, maintaining a virtual representation of the vehicular network alongside blockchain-secured FL to improve resilience to node failure [64]. The recurring insight across all three designs is that combining these mechanisms does more than add their individual benefits: the ledger gives FL a way to detect and exclude bad-faith contributions, while FL gives the ledger something meaningful to secure beyond simple transactions. The trade-off is architectural complexity.

The designs discussed above vary significantly in terms of how much of the consensus mechanism they define, and this transparency is informative. The double-layer design organizes data by importance rather than a specific protocol. Routine sensor data and speed information are stored on a vehicle-level chain and purged hourly, while trust values and safety messages are on a separate RSU-level chain. Each RSU evaluates every 30 s to see if it can append the next block. No Byzantine fault-tolerant protocol is defined for either layer, and only vehicles and RSUs participate, excluding edge or cloud tiers [61]. Two designs are notable for their explicit protocol details. The reputation-based design employs Practical Byzantine Fault Tolerance with an adjustment rule to calculate vehicle reputation scores, resulting in a latency reduction of 0.29 s and an increase in packet delivery ratio from 0.80 to 0.95 [62]. The blockchain-federated-Q-learning design uses a three-phase delegated protocol, recording only transaction records and content identifiers on-chain while keeping data off-chain. It distributes computation among local vehicles, roadside units, and a cloud server, achieving a consensus delay of 50 ms with an average end-to-end latency of 864 ms for a hundred-vehicle deployment [63]. One design incorporates a digital twin on a federated-blockchain architecture involving edge servers, roadside units, and vehicles, demonstrating faster convergence and resilience to failures, though it does not specify its consensus mechanism or latency [64]. Deployment varies, with some designs restricting participation to vehicles and infrastructure, while others include a cloud tier for global aggregation, ultimately influencing the design’s latency.

### 5.3. Lightweight and Resource-Aware Edge Security

Every mechanism discussed so far ultimately has to run on hardware far more constrained than a data-center server. A recent lightweight intrusion detection design combines a compact feature-fusion network with federated coordination, preserving detection quality within a small computational footprint [65]. A related sequential AI framework is built specifically for real-time operation in dynamic vehicular networks, prioritizing a fast, low-overhead architecture over larger models [66]. The insight running through this line of work is that resource-awareness must shape the architecture from the outset: a detector that is accurate but too slow or too demanding for its target hardware is not deployable. This resource ceiling is not incidental: the in-vehicle ECUs and roadside units that must host these detectors operate within power budgets far below those of the cloud servers on which many models are trained, so the feature-fusion, compression, and architectural choices made here determine whether a Category 2 mechanism is deployable at all rather than merely accurate in the laboratory. The same pressure returns in sharper form once large language models enter the picture in Section 6, where sheer model size, rather than coordination overhead, becomes the dominant constraint. Table 8 summarizes the Category 2 methods discussed across this section.

### 5.4. Category 2 Synthesis

One theme runs through this section: as security for the IoV shifts from a centralized detection model to an edge-based approach, each solution tends to create new challenges. While moving computation closer to the edge reduces latency, it also increases the number of decision points that need to be maintained and updated. FL relieves data-centralization but introduces a trust problem, since a shared model is only as reliable as the participants contributing to it. Blockchain addresses that trust problem but adds coordination overhead. Combining FL with blockchain addresses each mechanism’s weak point, at the cost of interdependent moving parts. All mechanisms must be aligned with the limited computational budget of real vehicles and roadside units. Importantly, none of the studies reviewed here examine these mechanisms in relation to adaptive adversaries who customize their updates according to the aggregation rules that are in use. Instead, the focus has primarily been on simple data poisoning. Additionally, the concerns regarding interpretability, which arise when decisions are made across numerous federated and blockchain-coordinated nodes, have not been addressed. These open questions carry directly into the Category 3 directions reviewed in Section 6.

## 6. Category 3: Large Language Models, Adversarial Robustness, and Explainable AI

The first two categories share an assumption worth making explicit: a detector is trustworthy because it performs well on held-out data, and the main challenge is scaling that detector across a growing, distributed network. Two loose ends from the preceding sections complicate this. The adversarial vulnerability note in Section 4.1 and the AI-specific threat landscape in Section 3.3 together make clear that strong reported accuracy does not guarantee resilience against a motivated attacker. Section 5’s closing observation notes that decentralizing detection across many nodes makes individual decisions harder, not easier, to inspect. Category 3 can be understood as a direct response to the gaps left by the other two categories, as well as introducing a capability they did not have: the ability to generate a natural-language account alongside a detection. This feature is just starting to be applied to vehicular security.

The evidence base for this category is more mixed than for the other two, and the distinction matters for how the findings below should be read. Of the ten studies scored in Table 11, four are evaluated on vehicular data and six on general IoT or enterprise traffic. Three further studies, discussed in Section 6.3 rather than tabulated, evaluate adversarial manipulation directly on CAN data, so that seven of the thirteen studies reviewed in this section rest on vehicular evidence. The eight foundational methods introduced below were developed and evaluated outside the vehicular setting entirely; they are included because the vehicular work builds on them, not as evidence about the IoV. The “Evaluated On” column of Tables 10 and 11 records the data used in every case, so that a method already demonstrated on vehicular traffic can be distinguished from one that remains a direction for IoV security rather than a validated result.

### 6.1. LLM-Driven Detection and Explanation

Vehicular network traffic is sequential and context-dependent, which is precisely the kind of structure transformer-based language models were built to exploit, so recent work explores intrusion detection as a language-modeling task over message sequences rather than solely as classification over fixed features. At the architectural level, one recent framework integrates large-language-model-driven analysis directly into a vehicle’s broader data pipeline alongside classical statistical, graph-based, and uncertainty-quantification components, validating the design across multiple CAN-bus datasets [14]. A complementary line of work forgoes a bespoke architecture in favor of representing constrained-device network traffic in text form so that a pretrained language model can be applied to anomaly detection without retraining the underlying transformer [33]. Together, these suggest that pretrained language models transfer usefully to network data, though neither fine-tunes a language model directly on vehicle-specific traffic, which is the gap closed by the work below.

Three capabilities are distinguished here, and each requires different evidence. Classification assigns a label and is evaluated by detection metrics on held-out data. Explanation generation produces a textual account of that label. The account itself is observable, but whether it reflects the factors that actually drove the decision is a separate question from whether it reads plausibly to a human reader [67]. Reasoning, in the stronger sense used here, is the ability to derive and justify a conclusion from context. A coherent step-by-step account is not evidence of it, since such an account need not represent the computation behind the prediction [68]. The studies reviewed below classify and generate rationales. None evaluates whether those rationales are faithful, so none provides evidence of reasoning in this sense.

The most direct evidence in a genuinely vehicular setting fine-tunes three language models on CAN-bus attack traffic and evaluates them both on classification and on their ability to produce a rationale through zero-shot prompting [32]. The classification is determined quantitatively, with the best model achieving near-perfect metrics on datasets using single attacks. The rationale is evaluated through specific examples rather than in a systematic manner. The authors note that the generated explanations incorrectly identify a CAN frame field and do not consistently use the correct field names. This serves as evidence that the coherence of an explanation does not necessarily correlate with its accuracy. A related design keeps an existing classifier in place and adds a multimodal language model purely as an explanatory layer, prompting it with the detector’s feature-attribution output to produce a narrative for a flagged CAN-bus message [69]. Detection there is performed by a gradient-boosted classifier rather than by the language model, and the narratives are evaluated by manual review and by a study in which ten researchers rated them on a five-point Likert scale for usability, relevance, fluency, accuracy, and overall satisfaction, which provides evidence about their perceived quality but does not by itself establish faithfulness to the detector’s internal decision process. Read together, the two works support the claim that language models are proving useful as an explanatory layer over a trained detector rather than as its replacement, and leave the stronger claim unevaluated.

When considered together, these four studies represent vehicular or network traffic as input for language models in significantly different ways, and this choice is crucial to the claims each system can ultimately make. The two studies discussed illustrate different uses of language models as explanatory tools. The first study’s vision-language design inputs a feature-attribution plot from the underlying classifier rather than the raw CAN frame, allowing the model to visualize another model’s reasoning [69]. In contrast, the second study’s encoder-only model tokenizes each CAN message into eleven components: timestamp, identifier, length, eight data bytes, and status, with hexadecimal fields converted to numeric values. Its decoder-only model receives the same message as a natural-language prompt asking if it is malicious [32]. Outside this vehicular evidence, the text-serialization approach represents each row of tabular flow data as a string concatenating each feature name with its value, so a pretrained model can process network traffic without a bespoke numerical encoder, and reports a throughput of 243.87 samples per second for its most efficient model on this representation [33], while the change-point framework converts language-model anomaly scores over a sliding window into a single evidence-accumulation statistic before thresholding, so its output is a change-point decision rather than a per-frame label [14]. None of the four studies specifies whether inference is conducted on the vehicle, at the roadside, or in the cloud. Given the significant computational cost mentioned earlier, deploying an undistilled model of this size in a vehicle is unrealistic. Therefore, these designs implicitly assume that inference will take place at the edge or in the cloud, rather than in the vehicle itself, despite not explicitly stating this.

Read against this vehicle-specific evidence, a broader comparative benchmark run on general IoT flow data, rather than vehicular traffic, offers a useful note of caution: a well-tuned gradient-boosted classifier can still match or exceed compact general-purpose language models on raw classification throughput [34]. The insight this contributes is that this category’s present advantage lies in contextual analysis and in the account it can produce, rather than in raw throughput, a distinction the vehicle-specific work above supports rather than contradicts, and one that motivates the discussion of lightweight and distilled deployment that follows.

A critical concern not addressed by any of the above works is the hallucination problem described in Section 2.2: an LLM-based detector can produce fluent, confident outputs that are factually incorrect. In vehicular security, a hallucinated benign classification for a genuine attack can suppress an automated safety response, and a hallucinated attack classification on normal traffic can trigger an unnecessary evasive maneuver. This risk must be treated as a first-class design constraint for any deployment, not an acknowledged limitation for future work. Possible mitigation strategies include calibrated uncertainty estimation, ensemble over multiple model samples, and output monitoring layers that flag low-confidence classifications for human review.

### 6.2. Lightweight and Distilled LLM Deployment for IoV

As outlined in Section 2.2, a forward pass of BERT-base on 128 tokens costs approximately 22.5 GFLOPs and takes about 342 ms on mobile-class hardware. This is a significant concern, as the budget per frame is less than one millisecond on a 500 kbit/s CAN bus. That gap, rather than parameter count alone, is why knowledge distillation rather than direct deployment has become the preferred approach in this line of work. Two additional constraints are specific to the vehicular setting and are not resolved by reducing model size. Autoregressive generation produces output token by token, so response time depends on the generated sequence length; none of the vehicle-specific studies reviewed in Section 6 reports end-to-end inference latency. When stochastic decoding is used, repeated inference can also produce different outputs for the same input, which can complicate reproducibility and verification of a security function. These considerations are relevant to the lifecycle-oriented cybersecurity engineering and validation processes addressed by ISO/SAE 21434 [70]. One early demonstration compresses a BERT-based language model into a lightweight architecture explicitly designed to preserve data locality on constrained IoT devices [35]. A more recent design goes further: rather than simply training a smaller model to imitate a larger one’s output, it introduces a ladder of intermediate connections between the large teacher network and a lightweight student built from efficient convolutional components, transferring structural knowledge at multiple levels rather than only at the output layer [71]. The shared insight is that the mechanism of compression matters as much as the size of the resulting model. The limitation common to both is that this line of research has so far been demonstrated on general IoT settings rather than on IoV data specifically, leaving open how much of a compressed model’s capacity can be surrendered before it stops recognizing vehicle-specific attack patterns. In effect, this transposes the resource-awareness lesson of Section 5 from the coordination cost of decentralized mechanisms to the raw capacity of the model itself: the same deployment ceiling now bites through parameter count rather than communication overhead.

### 6.3. Adversarial Robustness of ML-Based Security Models

#### 6.3.1. Attack Taxonomy

Adversarial attacks on ML-based IoV detectors can be classified along two orthogonal dimensions: the adversary’s access level and the attack objective.

In a *white-box* setting, the adversary has full knowledge of the model’s parameters and architecture, enabling gradient-based perturbation methods. In a *Grey-box* setting, the adversary knows the model’s architecture but not its parameters, and may exploit transferability of perturbations across models trained on the same data. In a *black-box* setting, the adversary can only query the model and observe outputs, requiring query-based optimization.

Let $f_{\theta }$ denote the detector under attack, with input $x\in X\subseteq R^{d}$ the feature encoding of a CAN frame or V2X message, true label y∈Y, and L(θ,x,y) the detector’s training loss (typically cross-entropy). An adversarial perturbation δ is restricted to an allowed set $S=\{\delta :∥\delta {∥}_{p}\leq \epsilon \}$, where ϵ is the perturbation budget and *p* is most commonly *∞* (bounding the largest single-field change) or 2 (bounding the perturbation’s overall magnitude); a feasible adversarial input further requires $x+\delta \in X_{\mathrm{valid}}$, the subset of X corresponding to protocol-valid CAN or V2X messages, as discussed below. The Fast Gradient Sign Method (FGSM) [9] produces δ in a single step, $x^{\prime }=x+\epsilon \cdot \mathrm{sign}\left(\nabla_{x}L\left(\theta ,x,y\right)\right)$; its simplicity makes it a standard baseline. Projected Gradient Descent (PGD) [72] strengthens this into a *T*-step iterative attack, $x^{t+1}=\Pi_{X_{\mathrm{valid}}⋂+\left(x+S\right)}\left(x^{t}+\alpha \cdot \mathrm{sign}\left(\nabla_{x}L\left(\theta ,x^{t},y\right)\right)\right)$ for t=0,…,T−1 with $x^{0}=x$, where α is the per-step size and $\Pi_{x+S}$ projects back onto the allowed perturbation set after each step; this produces significantly stronger attacks at the cost of more computation. Carlini & Wagner (C&W) [73] cast adversarial example generation as a constrained optimization problem, finding the minimum-norm perturbation that causes a misclassification, and has broken many defenses that were effective against FGSM and PGD. DeepFool [74] finds the closest decision boundary through an iterative linearisation of the classifier, producing minimal-norm perturbations useful for quantifying a model’s geometric robustness margin.

Many of these methods were originally developed and extensively evaluated for continuous, high-dimensional inputs, whereas a CAN frame is a discrete and tightly constrained representation. The identifier, the length code, and the eight payload bytes are discrete fields with fixed ranges, and a perturbed frame must remain protocol-valid to be accepted by a receiving ECU. The consequences are visible in the vehicular results themselves. Aloraini et al. [10] limit the allowed perturbations to the payload bytes and constrain values to the interval [0,255]. Within these restrictions, the FGSM and BIM prove to be the most effective attack strategies, achieving success rates of approximately 47% when each byte can change by at most one unit, and nearly 60% when the allowable change is increased to ten units. In contrast, the C&W and EAD methods do not succeed, as techniques that seek minimum-norm perturbations have limited flexibility when the feasible region is so narrow. Longari et al. [75] additionally round perturbed values to integers representable in the underlying bit vector, and observe both that this projection can impede convergence and that a successful evasion frequently leaves the payload indistinguishable from benign traffic, so that detection is avoided but the attack no longer achieves its purpose. A further difficulty is that the threat model is usually stated at the level of the detector’s feature vector rather than the wire: recent black-box work on CAN intrusion detection perturbs already preprocessed features, including timestamps and identifiers, without mapping the result back to a transmissible frame [76]. Neither of the studies above reports injection on a physical bus, and no comparable evaluation exists for V2X messages among the works reviewed here. Adversarial vulnerability in the IoV is therefore established in the feature space under stated domain constraints, while realizability on a bus or a wireless channel must be demonstrated separately for each representation and attack surface rather than assumed from the image domain.

The reviewed vehicular studies do not converge on a single robustness metric. Aloraini et al. [10] report robustness as the drop in F1-score under attack relative to the clean F1-score. Longari et al. [75] report the true positive rate (recall) of the detector under attack together with an aggregate perturbation (AP) measure, $\mathrm{AP}=\frac{1}{N}\sum_{i=1}^{N}{∥x_{i}-x_{i}^{\prime }∥}_{\infty }$, which quantifies the average magnitude of change needed to evade detection. Barletta et al. [76] instead report an attack success rate (ASR), the fraction of correctly classified inputs that an attack flips to an incorrect label, $\mathrm{ASR}=|\left\{i:f_{\theta }\left(x_{i}\right)=y_{i}\wedge f_{\theta }\left(x_{i}^{\prime }\right)\neq y_{i}\right\}\left|\;/\;\right|\left\{i:f_{\theta }\left(x_{i}\right)=y_{i}\right\}|$, alongside a weighted accuracy loss and an average perturbation norm. Robust accuracy (the post-attack value of whatever detection metric a study reports) and ASR are complementary rather than interchangeable: the first states how much detection capability survives an attack, and the second states how often the attack succeeds against inputs the detector originally classified correctly. Reporting both, together with the perturbation budget ϵ and norm *p* under which they were computed, is necessary for a result to be reproducible and comparable across studies, a requirement the works reviewed here meet only partially.

#### 6.3.2. Defences and Their Evaluation

Recent work in IoV responds to adversarial attacks less by proposing a single fix than by combining several partial defenses. One ensemble defense framework layers adversarial training, label smoothing, data augmentation, and a denoising autoencoder stage together, on the grounds that no individual defense mechanism is reliable enough in isolation [77]. A broader survey of generative adversarial networks (GANs) in intrusion detection reveals a double-edged insight: the same generative techniques capable of crafting convincing adversarial traffic can be turned defensively to synthesize realistic attack examples that improve a detector’s robustness during training [78], meaning adversarial robustness and generative modeling are becoming intertwined rather than opposing concerns.

Adversarial training and ensemble defenses provide empirical robustness but lack formal guarantees. Certified robustness methods provide provable bounds on model behavior under bounded perturbations. The most practically accessible technique is randomized smoothing [79], which constructs a certified classifier from an underlying base model by adding Gaussian noise to inputs at inference time and returning the plurality class across many samples; a certification radius can be derived analytically from the resulting output distribution. Interval bound propagation (IBP) provides an alternative by propagating worst-case perturbation bounds through each layer of a neural network analytically. Neither approach has yet been demonstrated for vehicular intrusion detection on realistic IoV data, representing a clear and tractable opportunity for future work.

The limitation that cuts across the entire adversarial robustness literature reviewed here is that evaluations test a defense against the same style of attack used to design it, rather than against a genuinely adaptive opponent. A real IoV adversary capable of observing how a detector responds would adjust their strategy accordingly. A PGD-adversarially-trained model evaluated only against PGD provides a narrower guarantee than it appears: an adaptive adversary who switches to C&W attacks may still succeed. Future work should evaluate defenses against an adaptive adversary who is given knowledge of the defense and optimizes accordingly. The AutoAttack framework [80] provides a practical methodology for this kind of evaluation.

### 6.4. Explainable AI for Trustworthy Detection

#### 6.4.1. Foundations and Design Tensions

A detector that reasons well, is efficiently deployable, and resists adversarial manipulation is still only as useful as an operator’s or a driver’s ability to trust its outputs. Explainability research provides tools for that trust.

*LIME* (Local Interpretable Model-agnostic Explanations) [81] approximates any classifier locally around a specific prediction using a sparse linear model fit on perturbed samples, producing a feature attribution that explains why a particular input was classified as it was. *SHAP* (SHapley Additive exPlanations) [82] derives feature attributions from cooperative game theory, assigning each feature a Shapley value that represents its average marginal contribution to the model’s output across all possible feature subsets. SHAP is grounded in Shapley-value attribution and provides a stronger theoretical basis than purely local surrogate fitting under its stated assumptions. Both are model-agnostic and have been applied to tabular network-traffic data with results that practicing analysts have found interpretable.

*Grad-CAM* (Gradient-weighted Class Activation Mapping) [83] is model-specific to convolutional architectures and produces a heat-map highlighting which spatial regions of an input most influenced a particular output. In the context of IoV, where convolutional detectors process sequences of packet headers or CAN-bus frames, Grad-CAM can highlight which time-steps within a sequence drove a detection—a local, temporally resolved explanation that complements the feature-level attributions from LIME and SHAP.

**Global versus local explanations.** A practical XAI system for IoV security arguably requires both *global* and *local* explanations [84]. Global explanations characterize which features matter most across the entire dataset, building a security analyst’s general understanding of what the model has learned. Local explanations justify individual alerts, providing the time-specific and sample-specific detail needed to act on a particular decision. A global explanation that says “message frequency is the most important feature” is necessary but not sufficient; a local explanation that says “this specific burst of CAN messages at timestamps $t_{1}$–$t_{3}$ drove the alarm” is actionable.

A known tension in XAI research is between *faithfulness*—whether an explanation accurately reflects the model’s internal reasoning—and *plausibility*—whether the explanation looks reasonable to a human expert. A LIME or SHAP attribution that highlights features a human expert would recognize as attack-relevant may not actually capture what the model learned; if the model relies on a spurious correlation, the explanation may appear plausible while faithfully reflecting a flawed detector. This has direct safety implications in IoV: an explanation that passes a human plausibility check can mask a model that would fail badly against inputs outside its training distribution. Evaluating faithfulness, rather than only plausibility, should be a standard requirement in IoV XAI work.

#### 6.4.2. Regulatory Context: EU AI Act

The EU AI Act [85], which came into force in 2024, classifies systems that directly determine the outcomes of safety functions, including automotive systems, as *high-risk AI*. The EU AI Act establishes transparency, information, record-keeping, human-oversight, and risk-management obligations for high-risk AI systems; depending on the system and applicable provisions, these requirements strengthen the need for traceable and interpretable security decisions. The ISO/SAE 21434 cybersecurity engineering standard for road vehicles [70] similarly emphasizes auditability and the ability to trace security decisions to their basis. These regulatory requirements mean that explainability for IoV security is not merely academically desirable: it is legally motivated and increasingly mandatory for European market access. Survey papers and research proposals in this space should frame XAI not as an optional enhancement but as a compliance requirement.

#### 6.4.3. Recent IoV-Specific XAI Work

A recent framework couples a global feature-selection stage with local, decision-specific explanations, giving an analyst two complementary views of the model’s behaviour [84]. A closely related design applies the same combination of a deep learning detector and an explainability layer directly to threat detection in connected-vehicle transportation data, demonstrating integration at the point of vehicular deployment rather than on generic benchmarks [86]. The limitation worth stating plainly is that an explanation is only as trustworthy as the detector producing it: explainability clarifies why a model reached a decision but does not verify that the decision was correct or that the model would resist adversarial manipulation. Robustness and explainability are complementary, not substitutes.

### 6.5. Cross-Category Comparison

Having examined each category in turn, it is useful to set them side by side along the dimensions that most affect deployment. Table 9 does so, synthesizing the data requirements, primary capability, privacy posture, adversarial robustness, explainability, inference cost, and regulatory alignment of all three categories. Reading across any single row shows how one property is treated under each dominant security-design problem: privacy, for example, is not addressed where the problem is detection effectiveness, becomes a core design objective where the problem is distributed deployment, and is addressed partially through local inference where the problem is assurance. The adversarial vulnerability and evaluation artifacts left open by Category 1 are answered by the robustness and explainability work of Category 3; the single-point-of-failure and scalability limits of centralized detection are answered by the decentralization of Category 2; and the coordination overhead and reduced inspectability that decentralization introduces in turn motivate the explainable, regulation-aware direction of Category 3. The comparison thus doubles as a map of the open gaps taken up in Section 7. The maturity rating in that table describes each category individually; whether the three have been combined within a single system is examined separately in Section 6.6.

### 6.6. Category 3 Synthesis

As shown in Table 10, the findings from this section indicate that this category is still developing. Unlike the first two categories, language-model-driven detection produces a natural-language account of a flagged message that an analyst can inspect, rather than a label alone; the accounts produced have so far been assessed for coherence and perceived usefulness rather than for faithfulness to the decision. However, each demonstration faces the same challenge: the significant computational requirements. Additionally, the risk of hallucination in safety-critical situations is not yet addressed as a critical design constraint, which it needs to be. Adversarial robustness has moved from demonstrating that detectors can be fooled toward combining multiple partial defenses and toward principled certified methods, but evaluations against adaptive adversaries remain absent. Explainability is beginning to distinguish between building general confidence in a model (global explanations) and justifying individual decisions (local explanations), and it has a clear regulatory mandate from the EU AI Act, but it remains a separate thread from robustness rather than an integrated one. None of the studies reviewed here addresses language-model-based contextual analysis, robustness, and explainability together within a single system evaluated on vehicular data, and, as Table 11 shows, none addresses even two of them; closing that separation is the opportunity this survey identifies.

Table 11 scores each of these studies against the three capabilities separately. The last two columns jointly constitute the explainability capability, which a study demonstrates only when the explanation it produces is also assessed. Read that way, three patterns are visible. First, no study demonstrates more than one of the three capabilities, so the absence identified here is not a near miss but a complete separation. Second, the adversarial column is empty for every study evaluated on vehicular data: the four studies that use captured in-vehicle traffic run no attack at all, while the single study that applies a full attack suite [77] uses neither a language model nor vehicular data. No detector in this corpus has been both trained on vehicular traffic and evaluated against a crafted perturbation. Third, the three capabilities are pursued by disjoint groups of studies, and every pairwise intersection is empty. This last pattern is what distinguishes the finding from the maturity rating in Table 9. A low maturity rating would predict few studies demonstrating all three capabilities; it would not predict that no study demonstrates even two, since pairs would be expected to arise by chance among independently developing lines of work. The study-level analysis therefore carries evidence that the aggregate maturity rating does not.

## 7. Future Research

Rather than restating limitations generically, this section treats each stated limitation as the starting point for a concrete research direction.

**Integrated language-aware, robust, and explainable systems.** Section 6 closed by noting that no reviewed work combines language-model-based contextual analysis, demonstrated adversarial robustness, and explainability, within a single system evaluated on vehicular data. Building such a system is a substantially harder engineering problem than advancing any one capability in isolation, since strengthening one can come at the expense of another: a heavily distilled model built for edge deployment may have less capacity for robust training or generating faithful explanations. Future work that treats these three properties as joint design objectives—rather than as separate lines of research to be combined later—would directly close the gap this survey identifies as most pressing.

**Standardized, vehicle-specific benchmarks and protocols.** Section 4.2 highlighted that de-duplication of a widely used benchmark substantially changes the apparent ranking of methods. Section 6.3 noted that adversarial defenses are typically evaluated against the same attack style used to design them rather than genuinely novel ones. Both observations point to the same underlying need: benchmarks and evaluation protocols built specifically for vehicular network security, with de-duplication, realistic class imbalance, temporal splits that prevent leakage, and adaptive adversarial evaluation treated as standard practice rather than author-level choices. Existing candidate datasets—the Car-Hacking Dataset [87], VeReMi [88], and CICIDS2017 [89]—each have known limitations (real-vehicle specificity, attack coverage, class balance) that a purpose-built IoV security benchmark would address. A community-agreed evaluation framework comparable to MLPerf in performance benchmarking would substantially improve comparability across papers.

**Scaling decentralized security without sacrificing coordination.** Section 5 traced a recurring pattern: each mechanism added to address one weakness introduces its own coordination or communication overhead. This pattern will continue as deployments grow to include more vehicles, more roadside infrastructure, and more cloud-edge tiers, unless future work treats coordination cost itself as a first-class design objective. Research that explicitly measures and minimizes the coordination burden introduced by each additional mechanism—rather than treating scalability as something to assume once a mechanism works at small scale—would make the decentralized designs surveyed here considerably more deployable. Hierarchical aggregation (where vehicles first aggregate locally within platoons or road segments before global aggregation) is a promising direction that is largely unexplored in the current vehicular FL literature.

**Adaptive defenses against adaptive adversaries.** Section 6.3 noted that no defense evaluated in this survey has been tested against an adversary that adapts specifically to the defense in place. Future work should explicitly model the attacker-defender interaction as a dynamic game rather than a one-shot evaluation, using frameworks such as AutoAttack [80] or adaptive evaluation protocols designed to discover a defense’s worst-case failure mode. This would give a more honest picture of deployment-time robustness and help prioritize which defenses are most worth investing in. Establishing that a constrained feature-space perturbation corresponds to a frame or message that an adversary can place on a CAN bus or V2X channel, while preserving the intended effect of the attack, would turn the assumed threat model into a demonstrated one.

**Regulatory alignment and standardization.** The EU AI Act [85] and ISO/SAE 21434 [70] together create a regulatory framework within which IoV security systems must operate. The EU AI Act requires high-risk AI systems to be transparent, auditable, and accompanied by technical documentation and human oversight mechanisms. ISO/SAE 21434 requires cybersecurity to be managed across the vehicle lifecycle, which on our reading extends to over-the-air updates that alter the behavior of an ML-based security component. Neither requirement is currently addressed by the methods reviewed in any category of this survey. Future work that explicitly designs for regulatory compliance–not as a post hoc consideration but as a primary design constraint–would be better positioned for commercial deployment, and would contribute a dimension of systems-level thinking that is currently absent from this literature.

**Integration with 5G/6G deployment constraints.** The security mechanisms discussed throughout this paper do not operate in isolation from the underlying communication infrastructure. Recent work on AI-assisted, 6G-enabled monitoring of the IoV illustrates this shift concretely, using intelligent reflecting surfaces together with AI-driven analysis to support secure monitoring under next-generation connectivity [90]. As 5G and early 6G infrastructure matures, it will ease some of the resource constraints that repeatedly surfaced across Section 4, Section 5 and Section 6, while introducing new architectural assumptions—sub-millisecond latency, network slicing, integrated sensing and communication—that the methods surveyed here have not yet been evaluated against. Future work that revisits detection, decentralized coordination, and language-aware, robust, and explainable security specifically under 5G/6G deployment assumptions would help ensure that the directions identified in this paper remain relevant as the underlying infrastructure changes.

## 8. Limitations of This Review

The evidence base assembled in Section 1.4 carries five limitations that bear on how the findings above should be read.

1. A single indexing service was queried. Relevant work published in venues that Scopus does not index, or in languages other than English, may not have been retrieved, and no claim of completeness is made.
2. Screening was purposive and proceeded in order of database relevance rather than exhaustively, which favors work that is well indexed and frequently cited. Backward and forward citation tracking from the retained studies was used to reduce this effect but does not remove it.
3. Screening and data extraction were carried out by the authors rather than by independent reviewers working in parallel, so the assignment of a study to a category rests on the authors’ reading of each source.
4. The corpus is smaller than a systematic review would assemble. The statements made here that a capability was not observed are therefore statements about the reviewed corpus rather than about the field as a whole.
5. Category 3 is the newest section of the corpus, and its studies are the least developed. Therefore, conclusions that are mainly based on these recent studies should be considered provisional until they are backed by independent replication and broader evaluation based on more vehicular evidence.

## 9. Conclusions

This survey examined machine-learning and deep-learning approaches to security in Internet-of-Vehicles networks, organizing the reviewed work into three categories, each defined by the dominant security-design problem it addresses. Each category contributes a distinct capability while exposing a limitation that motivates the next. Classical detectors (Section 4) report strong performance, but that performance is sensitive to evaluation choices and has not been tested against deliberately crafted inputs. Decentralized architectures (Section 5) relieve the scalability and single-point-of-failure concerns those detectors leave open, at the cost of coordination overhead and decisions that are harder to inspect. The third category (Section 6) responds to both: language-model-driven analysis moves beyond fixed pattern matching, recent adversarial defenses combine partial mechanisms rather than relying on one, and explainability gives operators a way to inspect otherwise opaque decisions under a clear regulatory mandate.

A study-level gap analysis shows that no reviewed work unites language-model-based contextual analysis, demonstrated adversarial robustness, and explainability in a single system evaluated on vehicular data, and that no vehicular detector in the corpus reports an adversarial evaluation at all. These observations should nevertheless be interpreted as provisional because several of the underlying studies were published only recently and have not yet been independently replicated across broader vehicular settings. That gap is what distinguishes this survey from prior work and what the research directions in Section 7 are aimed at closing. Taken together, the three categories and those directions offer researchers entering this area a structured map of what has been tried and where the open problems remain.

## Figures and Tables

**Table 1:** Comparison of existing surveys and the proposed work by specific machine-learning technique family. A family is marked **Covered** when the survey provides a dedicated discussion of methods in that family, **Partial** when it receives only brief or secondary mention, and **Not covered** when it does not appear in the survey.

Survey	Platform	Classical ML/DL	Federated Learning (FL) & Blockchain	Large Language Models	Adversarial Robustness	Explainable AI
[18]	Security (Deep Reinforcement Learning (DRL) for IoT IDS)	Covered	Not covered	Not covered	Partial	Not covered
[15]	Routing (UAV-assisted IoV)	Covered	Partial	Not covered	Not covered	Not covered
[19]	IDS (connected vehicles)	Covered	Partial	Not covered	Not covered	Not covered
[16]	Edge Computing (ITS)	Covered	Covered	Not covered	Not covered	Not covered
[8]	Security & Trust	Covered	Partial	Not covered	Not covered	Not covered
[17]	Security (SCI layers)	Covered	Covered	Not covered	Not covered	Not covered
**This survey**	**Security (IDS, edge, emerging)**	**Covered**	**Covered**	**Covered**	**Covered**	**Covered**

**Table 2:** Literature search, filtering, screening, and study selection process.

Stage	Query, Filter or Selection Step	Records
1	TITLE-ABS-KEY(("Internet of Vehicles" OR "IoV" OR "VANET" OR "vehicular network" OR "connected vehicle" OR "autonomous vehicle" OR "CAN bus" OR "V2X") AND ("security" OR "intrusion detection" OR "intrusion prevention" OR "attack detection" OR "misbehaviour detection" OR "misbehavior detection" OR "anomaly detection" OR "trust management") AND ("machine learning" OR "deep learning" OR "federated learning" OR "blockchain" OR "large language model" OR "LLM" OR "transformer" OR "adversarial" OR "explainable AI" OR "XAI" OR "interpretability")) OR TITLE-ABS-KEY(("vehicular" OR "automotive" OR "CAN bus" OR "V2X") AND ("large language model" OR "GPT" OR "BERT" OR "adversarial example" OR "adversarial robustness" OR "certified robustness" OR "SHAP" OR "LIME" OR "Grad-CAM"))	7315
2	Language restricted to English	7198
3	Document type restricted to article, conference paper and review	6288
4	Subject area restricted to computer science and engineering	6020
5	Records prioritized for title/abstract screening	170
6	Full-text assessment	60
7	Primary studies retained after eligibility assessment	34

**Table 3:** Intra-vehicle network protocols and their primary attack surfaces.

Protocol	Standard	Bandwidth	Typical Attacks
CAN bus	ISO 11898	1 Mbit/s	Fuzzy, replay, spoofing, bus-off
LIN	ISO 17987	20 kbit/s	Replay, spoofing
FlexRay	ISO 17458	10 Mbit/s	Bus flooding, message manipulation
Automotive Ethernet	IEEE 802.3	100 Mbit/s–1 Gbit/s	ARP spoofing, DoS, MitM
OBD-II	ISO 15765	–	Diagnostic command injection, firmware extraction

**Table 4:** Common network-layer security threats in IoV and the part of the network primarily affected.

Threat	Description	Primarily Affects
Spoofing/Sybil	Falsifying vehicle identity or position	Perception, V2X
Denial-of-Service	Exhausting communication or computing resources	V2X, edge
Eavesdropping/MitM	Intercepting or altering exchanged data	V2X communication
Black-hole/Gray-hole/Wormhole	Dropping or misdirecting forwarded messages	Routing
Data falsification	Injecting misleading sensor or application data	Application layer
CAN bus injection	Injecting forged frames onto the in-vehicle bus	Intra-vehicle
OBD-II exploitation	Abusing the diagnostic port to read or inject data	Intra-vehicle

**Table 5:** Criteria for the three-category classification. Categories are distinguished by their dominant security-design problem and principal level of intervention, rather than by algorithm family or publication period.

Dimension	Category 1	Category 2	Category 3
Dominant security-design problem	Detection effectiveness and attack discrimination	Distributed deployment, coordination, privacy, and trust	Security assurance under adversarial, opaque, or language-based detection
Principal intervention level	Detection model	System architecture	Detector assurance and behavior
Unit of analysis	Classifier and input data	Nodes, data, and coordination	Detector outputs, explanations, and adversarial behavior
Key unresolved limitation	Centralization, scalability, privacy, and robustness	Coordination complexity, transparency, and stronger robustness requirements	Deployment cost, output reliability, rationale faithfulness, and IoV validation
Representative technical strands	Tree ensembles, LSTM/GRU, CNN, stacked pipelines	Federated learning, blockchain-assisted trust, edge/hybrid deployment	Language-based AI, adversarial robustness, explainability/auditability

**Table 6:** Evaluation setting of the Category 1 studies. NR indicates that the source does not state the item.

Ref.	Dataset	Task	Validation Protocol	Imbalance Handling	De-Dup.	Metrics Reported and Aggregation
[45]	CICIDS2017; Car-Hacking	Multi-class, 6 and 5 classes	60/40 split with 5-fold CV (CICIDS2017); split ratio NR (Car-Hacking)	SMOTE with undersampling, applied to the training set only	Not applied	Accuracy, precision, recall, F1; per-class tables labelled weighted average, aggregation of the headline value NR
[46]	CICIoV2024	Multi-class, 6 classes	Stratified 80/20 split and 10-fold CV	None; natural distribution retained	Not applied	Accuracy, precision, recall, F1; macro-averaged
[47]	CICIoV2024	Multi-class, 5 classes survive de-duplication	Stratified *k*-fold, k=2	None; macro-averaging used instead	Applied; 99.75% of records removed	Accuracy, F1; macro-averaged, with fold standard deviation
[48]	Car-Hacking, OTIDS and Survival Analysis, merged	Binary and multi-class, 6 classes	80/20 split, no cross-validation	Random undersampling applied before the split	Not applied	Accuracy, precision, recall, F1 reported per class; no aggregate value, aggregation NR
[49]	CICIDS2017	NR	Stratified *k*-fold, *k* NR	SMOTE with undersampling	Not applied	Accuracy only, read from figures; no precision, recall or F1 reported
[50]	Private CAN capture; CICIDS2017	Multi-class, 9 and 7 classes	70/30 split with 10-fold CV on the training set	Variational-autoencoder synthesis and SMOTE, applied before the split	Not applied	Accuracy, precision, recall, F1; aggregation NR
[51]	V2AIX	Binary	Split reported without ratio	NR	NR	No classification metric reported; the stated figures come from an efficiency and privacy analysis

**Table 7:** Studies that share a benchmark, and what accounts for the divergence in their reported results. Acc. = detection accuracy; “–” indicates not reported.

Ref.	Year	Data Preparation	Task	F1 Aggregation	Acc. (%)	F1
*CICIoV2024, in-vehicle CAN. Same label space and same aggregation; the figures are comparable*						
[46]	2025	As distributed, 1,408,219 records	Multi-class, 6 classes	Macro	100	1.00
[47]	2026	De-duplicated to 3568 unique signatures, a 99.75% reduction	Multi-class, 5 classes survive	Macro	99.72	0.6415
*CICIDS2017, enterprise network. The figures are not comparable; the reasons are given in the columns*						
[45]	2023	As distributed; resampling applied to the training set only	Multi-class, 6 classes, DoS and DDoS merged	Not stated	99.90	0.9646
[50]	2025	Infiltration expanded from 36 to 10,000 records before partitioning	Multi-class, 7 classes	Not stated	99.83	0.9977
[49]	2025	Not reported	Not reported	No F1 reported	96.2	–

**Table 8:** Summary of decentralized and edge-assisted security methods reviewed in Category 2.

Ref.	Year	Core Mechanism	Evaluated On	Key Insight
[54]	2025	Deep Q-learning offloading with RSU and UAV load balancing	Simulation; no dataset	Energy is minimized under a latency constraint; the encryption layer is fixed and its overhead is measured rather than optimized
[55]	2025	Fused autoencoder, CNN and BiLSTM detector	UNSW-NB15; non-vehicular	Three-branch fusion reaches 99.59% AUC at 0.048 ms per sample; the federated component is proposed but not implemented
[56]	2023	Generic federated-systems taxonomy with an IoV IDS review	no experiments	Classifies federated systems on six dimensions, then compares fifteen IoV federated IDS studies across twelve criteria
[57]	2025	Personalized FL with a depthwise convolutional bottleneck	Car-Hacking, CAN-Train-Test and two enterprise sets	Per-vehicle personalization addresses the mismatch between a single global model and heterogeneous local traffic
[60]	2025	FedAvg-trained LSTM benchmarked against GRU and MLP	VeReMi; simulated V2X	LSTM reaches 96.75% across forty clients, but adding differential privacy reduces accuracy to 20.62%
[61]	2023	Vehicle and RSU blockchains with logistic-regression trust	SUMO and OMNeT++ simulation	An hourly-deleted vehicle chain holds low-value messages while a permanent RSU chain holds trust values, reducing vehicle storage load
[62]	2025	Additive-increase, multiplicative-decrease reputation on a permissioned chain	NS-3 simulation	Trust rises additively per honest exchange and falls multiplicatively on a false one, so misbehaviour is penalized faster than it is rehabilitated
[23]	2025	RSU-aggregated FL with a Transformer detector and top-*k* node selection	Car-Hacking and UNSW-NB15	Aggregating only the highest-accuracy local models and recording them on the ledger raises accuracy; no poisoning defence is evaluated
[63]	2024	Elliptic-curve encryption, federated Q-learning and off-chain storage	MATLAB; randomly generated traffic	Off-chain storage with delegated consensus lowers validation delay; the Q-learning component performs detection rather than decision-making
[64]	2025	Digital-twin participant prediction with reputation-weighted aggregation	CIFAR-10 over SUMO and NS-3 mobility	Predicting which vehicles stay connected accelerates convergence, while on-chain reputation weighting sustains accuracy under poisoning
[65]	2025	Transformer autoencoder and CNN-LSTM streams with feature-level aggregation	Car-Hacking	Feature representations rather than parameters are aggregated; the federated component is presented as a theoretical framework and is not evaluated
[66]	2026	Liquid neural network with correlation-based feature selection	Synthetic VANET set; 5000 records	Feature pruning ahead of a liquid neural network reduces reported detection latency to about 29 ms

**Table 9:** Cross-category comparison of the three IoV security categories reviewed in this survey. FL = federated learning; DP = differential privacy; N/A = not applicable.

Dimension	Cat. 1: Classical ML/DL	Cat. 2: Decentralized Edge	Cat. 3: LLM, Robustness, XAI
**Data requirement**	Centralized, labeled training data	Distributed local data; no raw sharing	Pre-trained model; minimal labeled IoV data for fine-tuning
**Primary capability**	Pattern-matching classification	Privacy-preserving collaborative learning	Contextual analysis and natural-language rationale generation
**Scalability**	Limited; centralized bottleneck	High; designed for distributed deployment	Moderate; constrained by model size
**Privacy**	Not addressed	Core design objective (FL, DP)	Partially addressed via local inference
**Adversarial robustness**	Not addressed	Partially (Byzantine-robust aggregation)	Active area; ensemble defences, some certified methods
**Explainability**	Limited (trees interpretable; DNNs opaque)	Poor; distributed models harder to inspect	Active area; LIME, SHAP, attention maps
**Inference cost**	Low–Moderate	Moderate (FL coordination overhead)	High (LLM); reduced by distillation
**Hallucination risk**	N/A	N/A	Present; must be mitigated in safety-critical use
**Regulatory alignment**	Not considered	Partially (data protection/GDPR)	EU AI Act and ISO 21434 directly relevant
**Maturity in IoV**	High; many benchmarked studies	Moderate; growing deployment literature	Low; mostly proof-of-concept
**Primary open limitation**	Adversarial vulnerability; evaluation artefacts	Coordination overhead; adaptive Byzantine attacks	Deployment cost; unbounded inference time; hallucination; rationale faithfulness unevaluated; limited IoV validation
**Representative techniques**	RF, XGBoost, LSTM, Convolutional Neural Network (CNN), stacked ensembles	FedAvg, FedProx, Krum, permissioned blockchain	GPT/BERT-based IDS, FGSM/PGD adversarial training, SHAP/LIME

**Table 10:** Summary of the Category 3 security methods reviewed in this survey.

Ref.	Year	Focus Area	Evaluated On	Key Insight
[32]	2025	LoRA fine-tuned language models for CAN-bus detection	Car-Hacking; in-vehicle CAN	Quantized LoRA fine-tuning brings billion-parameter models within reach for frame classification; the natural-language rationale is obtained by inference-time prompting and is illustrated rather than evaluated
[33]	2025	Fine-tuned BERT variants on text-serialized flows	RT-IoT2022; non-vehicular	Reports only loss convergence and inference speed on a one per cent sample; no accuracy, precision, recall or F1 is given
[69]	2025	Vision-language narration of feature-attribution output	Car-Hacking; in-vehicle CAN	Detection is performed by a gradient-boosted classifier and an unmodified multimodal model converts the resulting LIME plots into prose, assessed by manual review and a ten-participant survey
[34]	2026	Compact language models against classical baselines on network flows	CIC IoT 2023; non-vehicular	A tuned gradient-boosted classifier exceeds compact language models on F1, 96.96% against 96.18%, and runs roughly four orders of magnitude faster
[14]	2026	Language-model anomaly scores feeding an adaptive change-point detector	Four public CAN datasets	Window scores are conformally calibrated before detection; the Ethernet and V2X parts of the framework remain architectural
[35]	2024	Hash-encoded BERT trained from scratch	Edge-IIoTset; non-vehicular	Cryptographic hash encoding permits classification on untrusted servers, and a 16.7 MB model gives sub-second CPU inference
[71]	2025	Ladder-structured knowledge distillation	NSL-KDD and TON-IoT; non-vehicular	Structural knowledge is transferred at multiple levels rather than only at the output layer
[9]	2015	FGSM, foundational white-box attack	MNIST, CIFAR-10 and ImageNet; not vehicular	Single gradient step; defines the baseline against which IoV detectors should be evaluated
[72]	2017	PGD, iterative white-box attack	MNIST and CIFAR-10; not vehicular	Multi-step projected gradient descent; a harder attack baseline
[73]	2017	C&W, optimization-based white-box attack	MNIST, CIFAR-10 and ImageNet; not vehicular	Minimum-norm perturbation; breaks many empirical defences
[77]	2025	Ensemble adversarial defence	CICIDS2017 and CICIDS2018; enterprise	Combines adversarial training, label smoothing, data augmentation and a denoising autoencoder
[78]	2026	Review of generative adversarial networks in intrusion detection	Review; no experiments	Generative techniques threaten and strengthen detectors simultaneously
[79]	2019	Randomized smoothing for certified robustness	CIFAR-10 and ImageNet; not vehicular	Provides provable $\ell_{2}$ robustness certificates; not yet applied in the IoV
[80]	2020	AutoAttack, adaptive robustness evaluation	MNIST, CIFAR-10, CIFAR-100 and ImageNet; not vehicular	Parameter-free ensemble of attacks for reliable, adaptive evaluation of defences
[81]	2016	LIME, local model-agnostic explanations	Sentiment and newsgroup text, and image classifiers; not vehicular	Sparse linear local approximation; widely applicable to tabular network data
[82]	2017	SHAP, Shapley-value feature attributions	Synthetic tabular models and MNIST; not vehicular	Theoretically grounded; satisfies efficiency, symmetry and additivity properties
[83]	2017	Grad-CAM, gradient-based visual explanations	ILSVRC-15 and PASCAL VOC 2007; not vehicular	Highlights the time steps driving detection in convolutional IoV models
[84]	2025	Statistical and model-based feature selection with SHAP and LIME cross-checking	IP, IoT and 5G datasets; non-vehicular	Global scores prune features, then SHAP and LIME cross-check each local explanation, cutting explanation latency by 87 per cent
[86]	2025	SHAP-explained CNN-GRU detector	CICIoV2024; in-vehicle CAN	Post hoc attribution is applied to an offline detector; the authors list in-vehicle and edge deployment as future work

**Table 11:** Structured gap analysis of the ten Category 3 domain studies against the three capabilities whose joint absence this survey identifies. Vehicular studies are listed first. LM = language model.

Ref.	Evaluated On	LM Performs Detection	Adversarial Evaluation	Explanation Produced	Explanation Assessed
[32]	Car-Hacking; in-vehicle CAN	Yes	No	Yes	No
[69]	Car-Hacking; in-vehicle CAN	No	No	Yes	Yes
[14]	Four public CAN datasets	Yes	No	Partial	No
[86]	CICIoV2024; in-vehicle CAN	No	No	Yes	No
[33]	RT-IoT2022	Yes	No	No	No
[34]	CIC IoT 2023	Yes	No	No	No
[35]	Edge-IIoTset	Yes	No	No	No
[71]	NSL-KDD, TON-IoT	No	No	No	No
[77]	CICIDS2017, CICIDS2018	No	Yes	No	No
[84]	IP, IoT and 5G traces	No	No	Yes	Yes

## Data Availability

No new data were created or analyzed in this study. Data sharing is not applicable to this article.

## Abbreviations

The following abbreviations are used in this manuscript:

**AE**	Autoencoder
**ARP**	Address Resolution Protocol
**BERT**	Bidirectional Encoder Representations from Transformers
**CAN**	Controller Area Network
**C&W**	Carlini–Wagner (adversarial attack)
**CICIDS**	Canadian Institute for Cybersecurity Intrusion Detection Evaluation Dataset
**CNN**	Convolutional Neural Network
**DL**	Deep Learning
**DoS**	Denial-of-Service
**DP**	Differential Privacy
**DRL**	Deep Reinforcement Learning
**ECU**	Electronic Control Unit
**ECDSA**	Elliptic Curve Digital Signature Algorithm
**FGSM**	Fast Gradient Sign Method
**FL**	Federated Learning
**FPR**	False Positive Rate
**GAN**	Generative Adversarial Network
**GPT**	Generative Pre-trained Transformer
**Grad-CAM**	Gradient-weighted Class Activation Mapping
**GRU**	Gated Recurrent Unit
**IBP**	Interval Bound Propagation
**IDS**	Intrusion Detection System
**IIoT**	Industrial Internet of Things
**IoT**	Internet of Things
**IoV**	Internet of Vehicles
**IPS**	Intrusion Prevention System
**ISO**	International Organisation for Standardisation
**LIME**	Local Interpretable Model-agnostic Explanations
**LIN**	Local Interconnect Network
**LLM**	Large Language Model
**LSTM**	Long Short-Term Memory
**MEC**	Multi-Access Edge Computing
**MitM**	Man-in-the-Middle
**ML**	Machine Learning
**OBD**	On-Board Diagnostics
**OTA**	Over-the-Air
**OTIDS**	Offset-Ratio and Time-Interval-based Intrusion Detection System dataset
**PGD**	Projected Gradient Descent
**RF**	Random Forest
**RL**	Reinforcement Learning
**RSU**	Roadside Unit
**SHAP**	SHapley Additive exPlanations
**SMOTE**	Synthetic Minority Oversampling Technique
**V2I**	Vehicle-to-Infrastructure
**V2N**	Vehicle-to-Network
**V2P**	Vehicle-to-Pedestrian
**V2V**	Vehicle-to-Vehicle
**V2X**	Vehicle-to-Everything
**VANET**	Vehicular Ad hoc Network
**XAI**	Explainable Artificial Intelligence
**AI**	Artificial Intelligence
**XGBoost**	Extreme Gradient Boosting
